# Efficacy of Quadratus Lumborum Block for Pain Control in Patients Undergoing Hip Surgeries: A Systematic Review and Meta-Analysis

**DOI:** 10.3389/fmed.2021.771859

**Published:** 2022-02-03

**Authors:** Jinfeng Li, Chenpu Wei, Jiangfa Huang, Yuguo Li, Hongliang Liu, Jun Liu, Chunhua Jin

**Affiliations:** ^1^The Second Affiliated Hospital of Guangzhou University of Chinese Medicine (The Second Clinical Medical College of Guangzhou University of Chinese Medicine), Guangzhou, China; ^2^Applicants for Doctor Degree of Equivalent Level in Guangzhou University of Chinese Medicine, Guangzhou, China; ^3^Bone and Joint Research Team of Degeneration and Injury, Guangdong Provincial Academy of Chinese Medical Sciences, Guangzhou, China; ^4^Guangdong Second Traditional Chinese Medicine Hospital (Guangdong Province Engineering Technology Research Institute of Traditional Chinese Medicine), Guangzhou, China; ^5^The Fifth Clinical Medical College of Guangzhou University of Chinese Medicine, Guangzhou, China; ^6^Suzhou BenQ Medical Center, The Affiliated BenQ Hospital of Nanjing Medical University, Suzhou, China

**Keywords:** hip surgery, arthroplasty, arthroscopy, pain, nerve block

## Abstract

**Background:**

Several studies have reported the use of anterior, posterior and lateral quadratus lumborum block (QLB) for pain control in hip surgeries. However, high-quality evidence is lacking. The current review aimed to summarize data on the efficacy of QLB for pain control in patients undergoing hip surgeries.

**Methods:**

PubMed, Embase, and Google Scholar databases were searched up to August 5, 2021 for randomized controlled trials (RCTs) or non-RCTs assessing the efficacy of QLB for any type of hip surgery.

**Results:**

Thirteen studies were included (nine RCTs and four non-RCTs). On pooled analysis, there was a statistically significant reduction of 24-h total opioid consumption in patients receiving QLB as compared to the control group (MD: −9.92, 95% CI: −16.35, −3.48 *I*^2^ = 99% *p* = 0.003). We noted a statistically significant reduction of pain scores in the QLB group as compared to control group at 2–4 h (MD: −0.57, 95% CI: −0.98, −0.17 *I*^2^ = 61% *p* = 0.005), 6–8 h (MD: −1.45, 95% CI: −2.09, −0.81 *I*^2^ = 86% *p* < 0.00001), 12 h (MD: −1.12, 95% CI: −1.89, −0.34 *I*^2^ = 93% *p* = 0.005), 24 h (MD: −0.71, 95% CI: −1.27, −0.15 *I*^2^ = 89% *p* = 0.01) and 48 h (MD: −0.76, 95% CI: −1.37, −0.16 *I*^2^ = 85% *p* = 0.01) after the procedure. There was a statistically significant reduction in the risk of nausea/vomiting (RR: 0.40, 95% CI: 0.18, 0.88 *I*^2^ = 62% *p* = 0.02) in patients receiving QLB but no difference in the risk of pruritis (RR: 0.46, 95% CI: 0.17, 1.24 *I*^2^ = 16% *p* = 0.13) and urinary retention (RR: 0.44, 95% CI: 0.19, 1.02 *I*^2^ = 0% *p* = 0.06).

**Conclusion:**

QLB as a part of a multimodal analgesic regimen reduces opioid consumption and pain scores in patients undergoing hip surgeries. The certainty of evidence based on GRADE was moderate. Despite the statistically significant results, the clinical relevance of the analgesic efficacy of QLB is debatable due to the small effect size.

**Systematic Review Registration:**

https://www.crd.york.ac.uk/prospero/, identifier: CRD42021267861.

## Introduction

Surgical intervention of the hip joint is frequently used in adolescents with congenital hip dysplasia, adults and elderly patients requiring arthroscopy or joint replacement surgeries (1). Early physiotherapy and rehabilitation after hip surgery are highly beneficial, and early mobilization is known to improve outcomes (2, 3). However, post-operative pain is an important limiting factor that can slow down the rehabilitation process. Despite extensive research, the most optimal analgesic regime for hip surgeries is still debatable (4, 5). Opioids are the most common drugs used for pain control in the post-operative period, albeit with several side effects like nausea, vomiting, dizziness, and urinary retention (6). As the elderly constitute a significant proportion of patients undergoing hip surgeries, respiratory depression and sedation with opioids can lead to significantly prolonged recovery (7).

In this context, several regional analgesic techniques have been described to manage pain after hip surgeries. Local anesthetic infiltration of the hip joint, either as single-shot injections or continuous infiltration *via* catheters, has been reported to be ineffective for pain control after arthroplasty surgeries (8, 9). On the other hand, nerve blocks like the femoral nerve block, fascia iliaca block, lumbar plexus block are known to improve pain scores and reduce analgesic consumption after hip surgeries. However, they can also result in muscle weakness which can increase the incidence of falls (4). Furthermore, lumbar plexus block can result in serious adverse events like risk of high neuraxial anesthesia, hypotension, and local anesthetic toxicity (4). Alternatively, epidural anesthesia can provide good pain relief in hip surgery patients but is associated with technical difficulties, side effects like hypotension and headaches, and motor weakness. A modification of the classical technique, the walking epidural anesthesia has shown to improve motor control but could be associated with reduced pain control (4, 5).

In an attempt to find a better regional analgesic technique, several studies evaluated the use of quadratus lumborum block (QLB) for pain control in hip surgeries (10, 11). The QLB was first described by Blanco in 2007 and is an interfascial plane block of the posterior abdominal wall (12). The key anatomical landmark involved with the block is the quadratus lumborum muscle and the thoracolumbar fascia (TLF). The TLF is composed of aponeuroses and fascia layers which encloses the back muscles and connects the anterolateral abdominal wall with the lumbar paravertebral region. While the exact mechanism of QLB is unclear, it is postulated that the spread of local anesthetic along the TLF in to the paravertebral space leads to the analgesic action. With time, the approach has evolved into three distinct types with local anesthetic being deposited posterior, anterior, or lateral to the quadratus lumborum muscle (13). In the anterior QLB, local anesthetic is deposited in front of the quadratus lumborum muscle at the level of its attachment to the transverse process of L4 vertebra and the drug spreads between the quadratus lumborum and the psoas major muscle. In the posterior type, the anesthetic is deposited posteriorly between the quadratus lumborum and the medial lamina of TLF while in the lateral block, the medication is injected lateral to the quadratus lumborum muscle in the region of its contact with the transversalis fascia. All three types of QLBs have been used for a variety of indications and to date, the exact mechanism of action of the three types of blocks and their specific indications are still unclear (13–15).

Research indicates that QLB can successfully manage post-operative pain in patients undergoing abdominal surgeries, renal surgeries, and cesarean sections (14), but its efficacy for pain control after hip surgeries is still unclear. Since previous review articles on QLB could include only a limited number of studies on hip surgery (14, 15), there is a need for a comprehensive systematic review assessing the efficacy of QLB in patients undergoing hip surgeries. The main aim of the current study was to systematically search literature and pool evidence on the effectiveness of QLB in reducing analgesic consumption and improving pain scores in patients undergoing hip surgeries.

## Materials and Methods

The current study was conducted as per the guidelines of the PRISMA statement (Preferred Reporting Items for Systematic Reviews and Meta-analyses) (16) and the Cochrane Handbook for Systematic Reviews of Intervention (17) (Supplementary Table 1). We registered the study on PROSPERO (CRD42021267861).

### Literature Search

We designed a systematic search strategy with the aid of the medical librarian to explore the electronic databases, such as PubMed, Embase, and Google Scholar for relevant articles. The search limits were set from inception to August 5, 2021, with no language restrictions. The keywords used for the literature search included: “Quadratus lumborum block,” “Regional anesthesia,” “Hip surgery,” “Hip fracture,” “Hip arthroplasty,” and “Hip arthroscopy.” Details of the literature search common to all databases are presented in Supplementary Table 2. Two reviewers carried out the electronic search independent of each other. The primary search results were assessed initially by their titles and abstracts to identify citations requiring full-text analysis. The full texts of the articles were reviewed by the two reviewers independently based on the inclusion and exclusion criteria. Any disagreements were resolved by discussion. We also carried out manual scoping of the bibliography in included studies for any additional articles.

### Inclusion Criteria

Eligibility criteria for this review were structured using the PICOS (Population, Intervention, Comparison, Outcome, and Study design) framework. Details are as follows:

*Population*: Patients undergoing any type of hip surgery
*Intervention*: QLB administered by any approach (anterior/posterior)
*Comparison*: No block or sham block with normal saline
*Outcomes*: Post-operative analgesic consumption, and/or pain scores, and/or adverse events.
*Study design*: Randomized controlled trials (RCTs), Controlled clinical trials (CCTs), and retrospective studies

Exclusion criteria were: (1) Studies comparing QLB with another nerve block. (2) Studies not specifically on hip surgeries. (3) Studies not reporting relevant outcomes. (4) Abstracts, editorials, review articles, and case reports.

### Data Extraction and Risk of Bias Assessment

A data extraction sheet was used by two reviewers to extract relevant data from the studies. Details of the first author, publication year, study location, study type, surgery type, sample size, age and gender details, duration of surgery, the protocol of QLB, post-operative analgesic drugs, and study outcomes were extracted. The outcomes of interest for our review were 24-h analgesic consumption in morphine equivalents, pain scores at rest on the Visual Analog Scale (VAS), and adverse events. A descriptive analysis was carried out for studies not reporting data amenable for the meta-analysis.

Two reviewers independently assessed the quality of included RCTs using the Cochrane Collaboration's risk of bias assessment tool-2 (17). Every study was assessed for randomization process, deviation from intended intervention, missing outcome data, measurement of outcomes, and selection of reported results. Based on the risk of bias in individual domains, the overall bias was marked as “high risk,” “some concerns,” or “low risk.” For non-RCTs, the risk of a bias assessment tool for non-randomized studies (RoBANS) was used (18). Studies were assessed for: selection of participants, confounding variables, intervention measurements, blinding of outcome assessment, incomplete outcome data, and selective outcome reporting. Any disagreements related to data extraction or quality assessment were resolved by discussion. The certainty of the evidence was assessed using the Grading of Recommendations Assessment, Development, and Evaluation (GRADE) tool using the GRADEpro GDT software [GRADEpro Guideline Development Tool. McMaster University, 2020 (developed by Evidence Prime, Inc.)].

### Statistical Analysis

The “Review Manager” software [RevMan, version 5.3; Nordic Cochrane Center (Cochrane Collaboration), Copenhagen, Denmark; 2014] was used for the meta-analysis. Total analgesic consumption in morphine equivalents and pain scores at different time intervals were expressed as the mean difference (MD) with 95% confidence intervals (CI). In case studies that used any other opioid in the post-operative period, data was converted into morphine equivalents for this analysis (19). For graphical data, Engauge Digitizer Version 12.1 was used to extract numerical data. Median, range and interquartile range data was converted into the mean and standard deviation (SD) when required using the method of Wan et al. (20). Data on adverse events were pooled using risk ratios (RR). The random-effects model was used for all the meta-analyses. A sub-group analysis was conducted based on the study type. We also conducted a sensitivity analysis to assess if any study had an undue influence on the overall results of total analgesic consumption and pain outcomes. Each study was sequentially excluded in the meta-analysis software to recalculate the total effect size. To explore the cause of heterogeneity, we conducted a meta-regression analysis for the outcomes of 24 h total analgesic consumption, 12 and 24 h pain scores. Covariates included were age, male gender, sample size, type of QLB block and type of local anesthetic. Meta-regression was not conducted for other outcomes due to limited number of studies. The analysis was conducted using Open MetaAnalyst software. An important limitation of the software is the unavailability of *R*^2^-values which, therefore, could not be reported in our analysis.

Heterogeneity was assessed using the *I*^2^ statistic. *I*^2^-values of 25–50% represented low, values of 50–75% medium, and more than 75% represented substantial heterogeneity. Funnel plot was used to assess publication bias. *P* < 0.05 was considered statistically significant.

## Results

### Details of Included Studies

**Figure 1:**
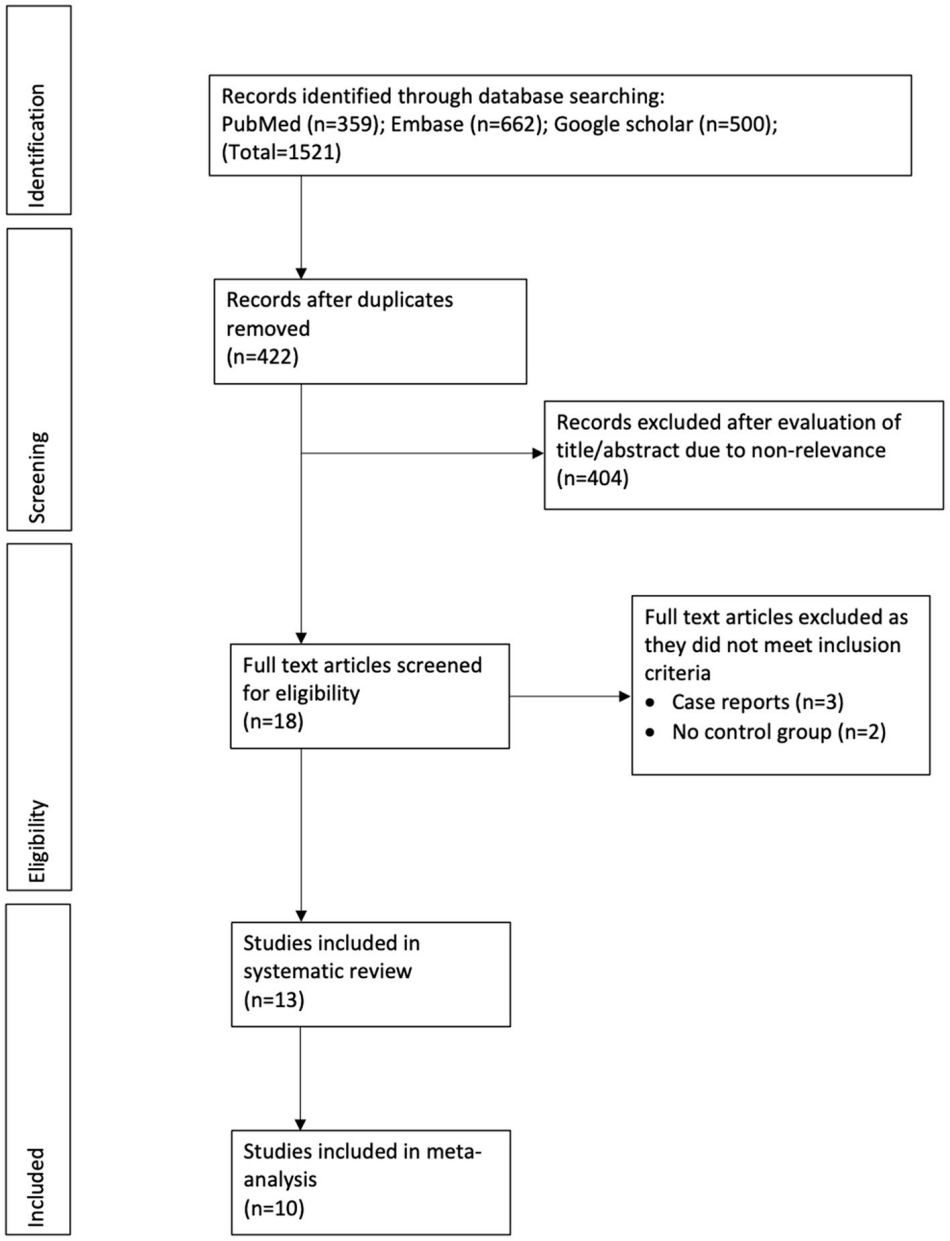
Study flow chart.

The number of search results at each stage is summarized in Figure 1. Eighteen articles were selected for full-text analysis. Of them, thirteen studies fulfilled the inclusion criteria and were analyzed in this review (21–33).

**Figure 2:**
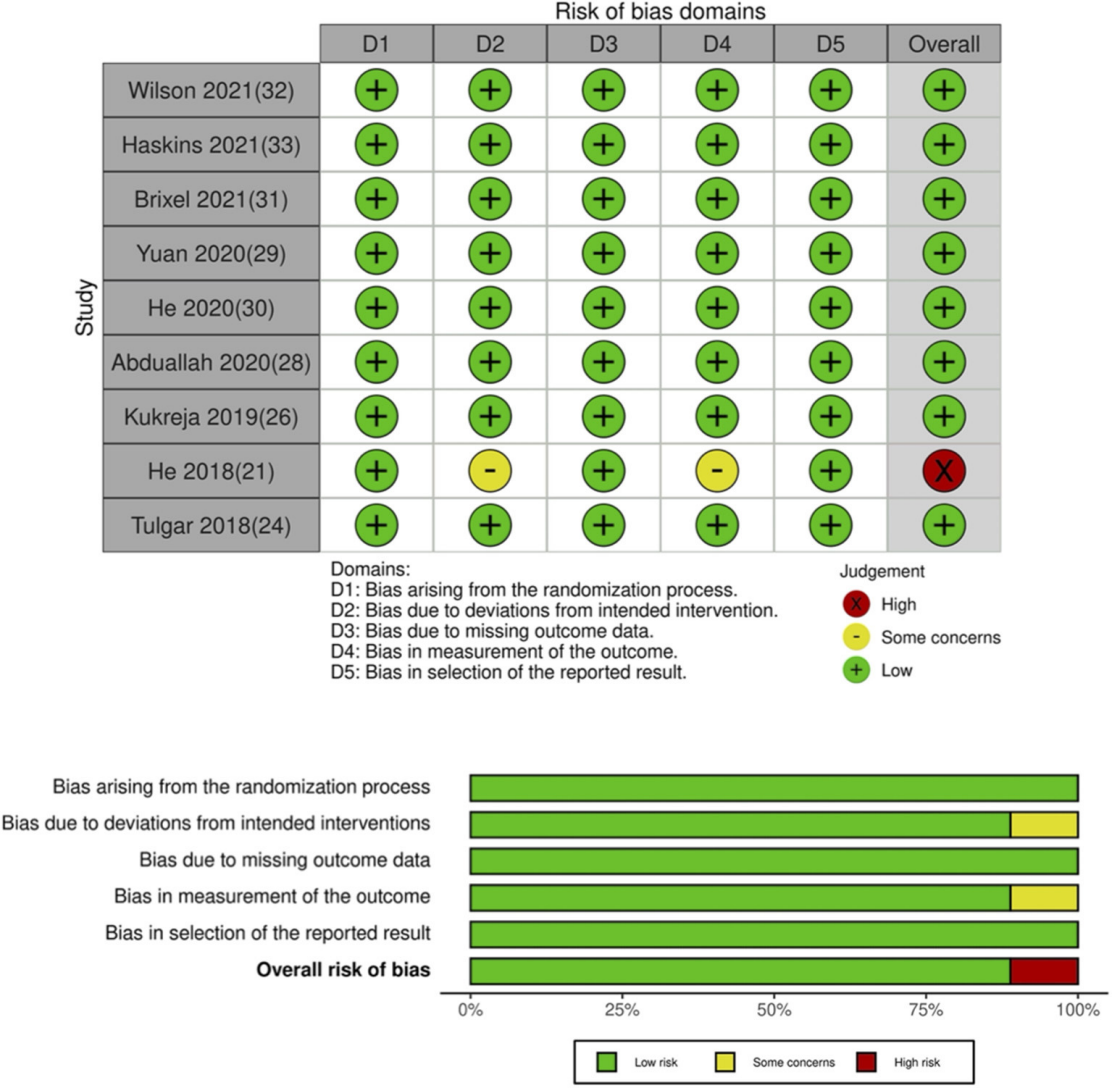
Risk of bias plot for RCTs.

**Figure 3:**
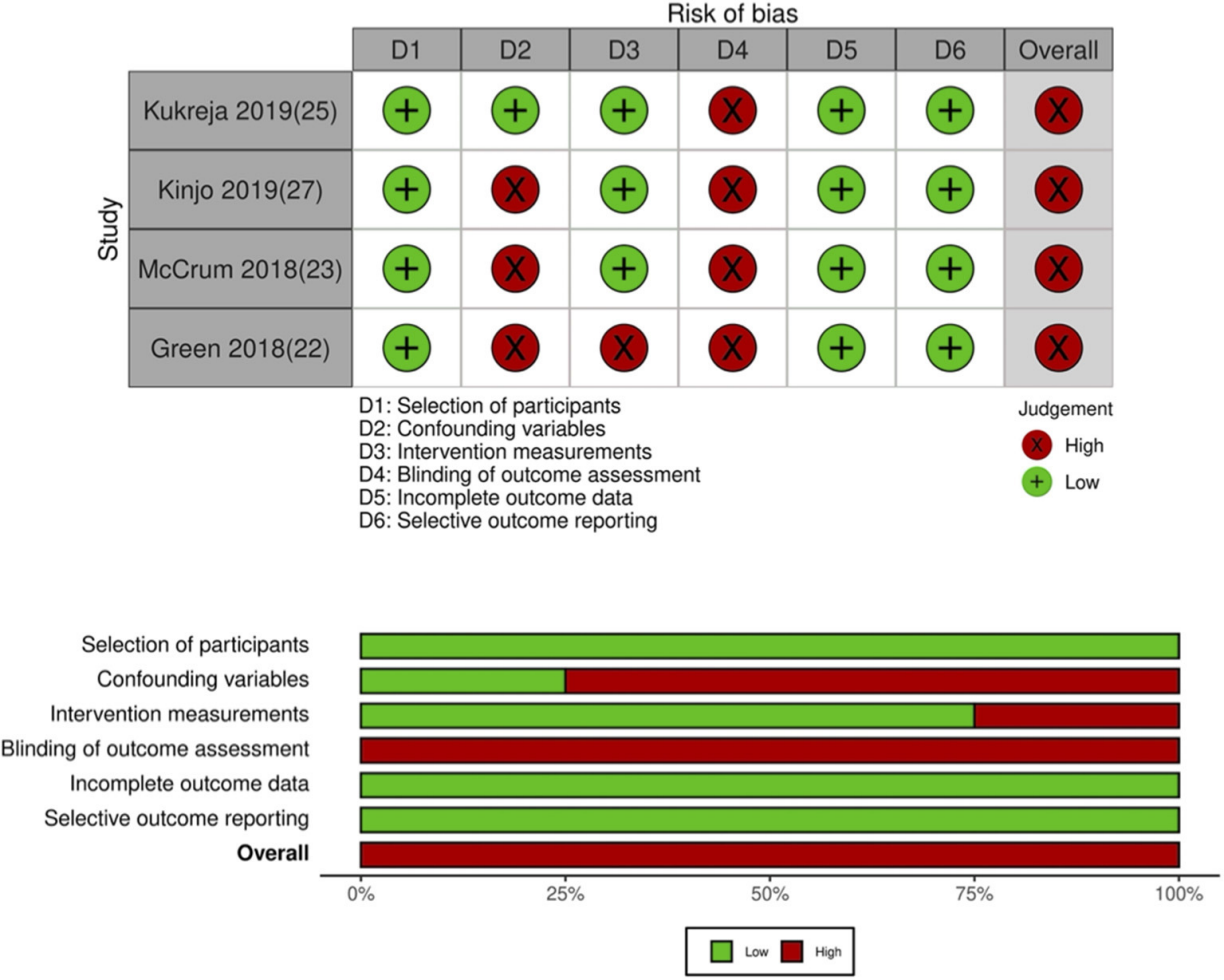
Risk of bias plot for non-RCTs.

Details of the included studies are presented in Table 1. Nine (21, 24, 26, 28–33) were RCTs while four (22, 23, 25, 27) were retrospective studies. Most studies were carried out in the USA or China. Seven (21, 22, 25, 26, 28, 30, 31) were conducted on patients undergoing hip arthroplasty, while five (23, 27, 29, 32, 33) were on patients undergoing arthroscopic surgeries. The sample size of the QLB group ranged from 10 to 79 patients while that of the control group ranged from 10 to 159 patients. QLB block was administered pre-operatively in all studies except for two RCTs (21, 28) wherein the block was administered after the surgical procedure. Four studies used the anterior approach while one study (32) used the lateral approach for QLB. Ropivacaine was the most common local anesthetic used followed by bupivacaine. The post-operative analgesic protocol differed across the included studies. The authors' judgment of the overall quality of included RCTs is presented in Table 2. Except for the trial of He et al. (21) which has a high risk of bias, all other trials were of high quality with low risk of bias (Figure 2). Quality assessment of non-RCTs is presented in Table 3. The majority of studies had a high risk of bias for confounding factors. Expectedly, all studies had a high risk of bias for blinding of outcome assessment (Figure 3).

**Table 1:** Details of included studies.

**References**	**Type**	**Location**	**Type of surgery**	**Protocol for nerve block**	**Sample size**		**Mean/median age (years)**		**Male gender (%)**		**Surgery duration (min)**		**Post-operative analgesic drugs**
					**QLB**	**Control**	**QLB**	**Control**	**QLB**	**Control**	**QLB**	**Control**	
Wilson et al. (32)	RCT	USA	Arthroscopy	Pre-operative USG guided lateral QLB with 40 ml of 0.25% ropivacaine	22	24	29.8	37.1	36.4	41.7	149.9 ± 53.1	163.6 ± 51.1	Oxycodone, IV hydromorphone
Haskins et al. (33)	RCT	USA	Arthroscopy	Pre-operative USG guided anterior QLB with 30 ml of 0.5% bupivacaine and 2 mg of dexamethasone	48	48	36	36	50	45.8	95 ± 23	95 ± 33	Oxycodone, PCM and naproxen/indomethacin and IV hydromorphone as rescue analgesic
Brixel et al. (31)	RCT	France	Arthroplasty	Pre-operative USG guided posterior QLB with 30 ml of 0.33% ropivacaine	50	50	68	65	60	40	70 (63–77)	68 (61–81)	IV PCM, IV ketoprofen, and morphine PCA
Yuan et al. (29)	RCT	China	Arthroscopy	Pre-operative USG guided posterior QLB with 0.4% ropivacaine	40	40	36.7	36.5	47.4	50	80.8 ± 8.1	82.5 ± 9.3	Sufentanil PCA, oral PCM and diclofenac
He et al. (30)	RCT	China	Arthroplasty	Pre-operative USG guided posterior QLB with 0.33% ropivacaine	44	44	66	67	29.5	25	98 ± 8	100 ± 7	IV parecoxib, oral PCM, and morphine PCA
Abduallah et al. (28)	RCT	Egypt	Arthroplasty	Post-operative USG guided posterior QLB with 30 ml of 0.25% bupivacaine	30	30	67.9	66.4	30	36.7	122 ± 9.2	125 ± 4.6	IV PCM and morphine rescue analgesic
Kukreja et al. (25)	R	USA	Arthroplasty	Pre-operative USG guided posterior QLB with 20 ml of 0.25% bupivacaine	79	159	NR	NR	NR	NR	NR	NR	NR
Kukreja et al. (26)	RCT	USA	Arthroplasty	Pre-operative USG guided anterior QLB with 30 ml of 0.25% bupivacaine	36	35	58.6	58	44.4	57.1	NR	NR	NR
Kinjo et al. (27)	R	USA	Arthroscopy	Pre-operative USG guided anterior QLB with 20–30 ml of 0.33–0.5% ropivacaine	15	54	35	35	33	55	97 ± 22.6	89 ± 20.1	NR
Tulgar et al. (24)	RCT	Turkey	Hip and proximal femur surgery	Pre-operative USG guided posterior QLB with 20 ml of 0.5% bupivacaine and 10 ml of 2% lidocaine	20	20	68.7	68.7	25	25	107 ± 19.9	110 ± 21.3	IV PCM and tramadol PCA, IV fentanyl and IM diclofenac as rescue analgesic
McCrum et al. (23)	R	USA	Arthroscopy	Pre-operative USG guided anterior QLB with 20–30 ml of 0.5% ropivacaine, 20–30 mcg of dexmedetomidine and 4 mg dexamethasone	28	28	37	36	39.3	28.6	74 ± 34	69 ± 20	IV PCM, IV ketorolac, and IV hydromorphone as rescue analgesic
He et al. (21)	RCT	China	Arthroplasty	Post-operative USG guided posterior QLB with 30 ml of 0.33% ropivacaine	30	30	64	65	46.7	43.3	91 ± 21	96 ± 19	Sufentanil PCA
Stuart Green et al. (22)	R	USA	Arthroplasty	Pre-operative USG guided QLB with 30 ml of 0.5% ropivacaine	10	10	NR	NR	NR	NR	NR	NR	NR

*IV, intravenous; IM, intramuscular; R, Retrospective; RCT, randomized controlled trial; PCM, paracetamol; PCA, patient controlled analgesia; NR, not reported; QLB, quadratus lumborum block; USG, ultrasound*.

**Table 2:** Risk of bias in included RCTs.

**References**	**Randomization process**	**Deviation from intended intervention**	**Missing outcome data**	**Measurement of outcomes**	**Selection of reported result**	**Overall risk of bias**
Wilson et al. (32)	Low risk	Low risk	Low risk	Low risk	Low risk	Low risk
Haskins et al. (33)	Low risk	Low risk	Low risk	Low risk	Low risk	Low risk
Brixel et al. (31)	Low risk	Low risk	Low risk	Low risk	Low risk	Low risk
Yuan et al. (29)	Low risk	Low risk	Low risk	Low risk	Low risk	Low risk
He et al. (30)	Low risk	Low risk	Low risk	Low risk	Low risk	Low risk
Abduallah et al. (28)	Low risk	Low risk	Low risk	Low risk	Low risk	Low risk
Kukreja et al. (26)	Low risk	Low risk	Low risk	Low risk	Low risk	Low risk
He et al. (21)	Low risk	Some concerns	Low risk	Some concerns	Low risk	High risk
Tulgar et al. (24)	Low risk	Low risk	Low risk	Low risk	Low risk	Low risk

**Table 3:** Risk of bias in included non-RCTs.

**References**	**Selection of participants**	**Confounding variables**	**Intervention measurements**	**Blinding of outcome assessment**	**Incomplete outcome data**	**Selective outcome reporting**	**Overall risk of bias**
Kukreja et al. (25)	Low risk	Low risk	Low risk	High risk	Low risk	Low risk	High risk
Kinjo et al. (27)	Low risk	High risk	Low risk	High risk	Low risk	Low risk	High risk
McCrum et al. (23)	Low risk	High risk	Low risk	High risk	Low risk	Low risk	High risk
Stuart Green et al. (22)	Low risk	High risk	High risk	High risk	Low risk	Low risk	High risk

### Meta-Analysis

**Figure 4:**
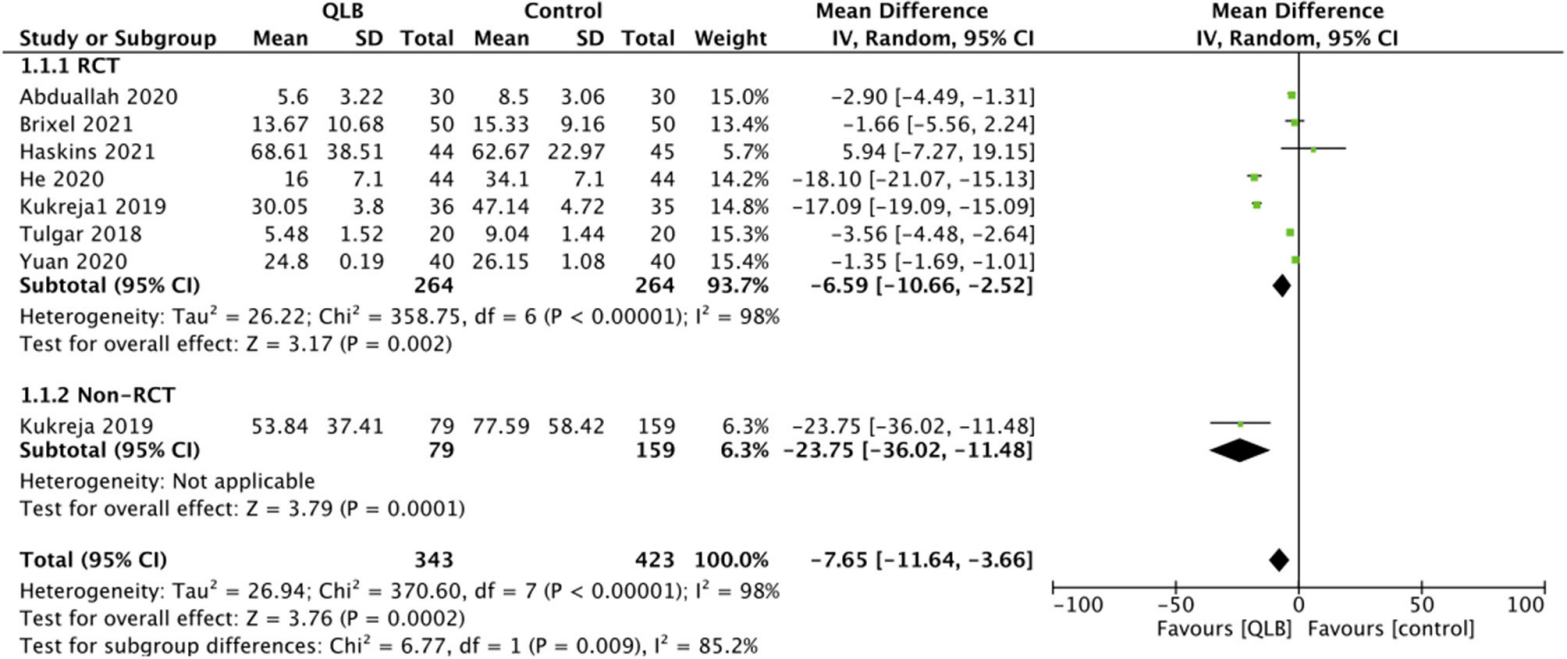
Meta-analysis of 24-h total analgesic consumption between QLB and control groups with sub-group analysis based on study type.

Eight studies reported data on 24-h total analgesic consumption. On pooled analysis, there was a statistically significant reduction of opioid consumption in morphine equivalents in patients receiving QLB as compared to controls (MD: −7.65, 95% CI: −11.64, −3.66 *I*^2^ = 98% *p* = 0.0002) (Figure 4). There was no evidence of publication bias on visual inspection of funnel plot (Supplementary Figure 1). The results did not change on exclusion of any study during a sensitivity analysis. Sub-group analysis of RCTs (MD: −6.59, 95% CI: −10.66, −2.52 *I*^2^ = 98% *p* = 0.002) and the lone non-RCT (MD: −23.75, 95% CI: −36.02, −11.48 *p* = 0.0001) demonstrated similar results (Figure 4). On excluding two studies on arthroscopy (29, 33), meta-analysis of RCTs reporting only arthroplasty results revealed significantly reduced opioid consumption in the QLB group (MD: −8.67, 95% CI: −14.90, −2.44 *I*^2^ = 98% *p* = 0.006) (Supplementary Figure 2).

**Figure 5:**
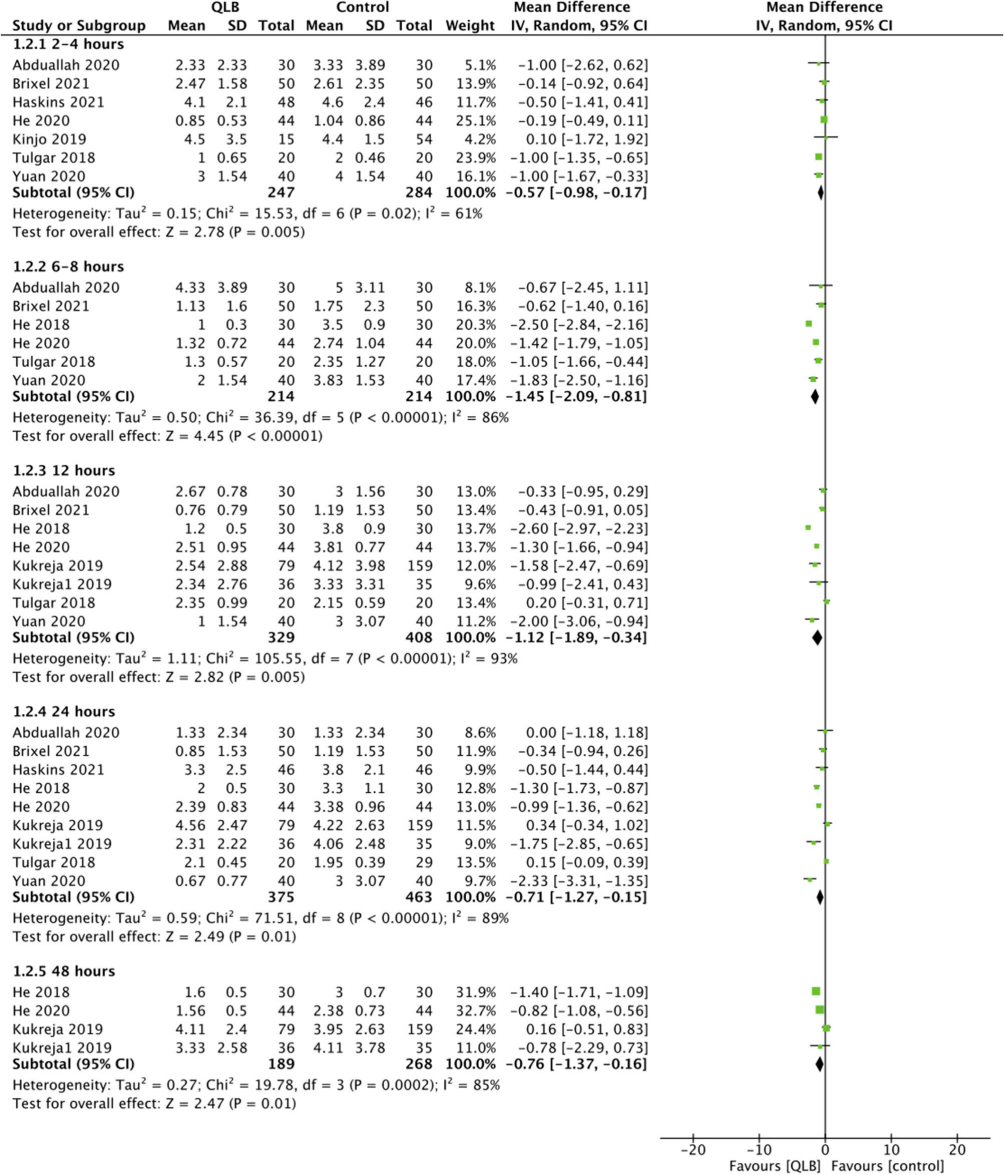
Meta-analysis of pain scores between QLB and control groups with sub-group at different time points.

On meta-analysis of pain scores at rest, we noted a statistically significant reduction of pain scores in the QLB group at 2–4 h (MD: −0.57, 95% CI: −0.98, −0.17 *I*^2^ = 61% *p* = 0.005), 6–8 h (MD: −1.45, 95% CI: −2.09, −0.81 *I*^2^ = 86% *p* < 0.00001), 12 h (MD: −1.12, 95% CI: −1.89, −0.34 *I*^2^ = 93% *p* = 0.005), 24 h (MD: −0.71, 95% CI: −1.27, −0.15 *I*^2^ = 89% *p* = 0.01) and 48 h (MD: −0.76, 95% CI: −1.37, −0.16 *I*^2^ = 85% *p* = 0.01) as compared to control group (Figure 5). On exclusion of two retrospective studies in the analysis (25, 27), the results did not change significance and there was a statistically significant reduction of pain scores at 2–4 h (MD: −0.60, 95% CI: −1.02, −0.18 *I*^2^ = 67% *p* = 0.005), 6–8 h (MD: −1.45, 95% CI: −2.09, −0.81 *I*^2^ = 86% *p* < 0.0001), 12 h (MD: −1.05, 95% CI: −1.91, −0.20 *I*^2^ = 94% *p* = 0.02), 24 h (MD: −0.85, 95% CI: −1.45, −0.25 *I*^2^ = 89% *p* = 0.006) and 48 h (MD: −1.07, 95% CI: −1.57, −0.57 *I*^2^ = 75% *p* < 0.0001) in patients receiving QLB as compared to controls (Supplementary Figure 3). During the sensitivity analysis, exclusion of the study of Yuan et al. (29) from the 24 h pain score changed the significance of the results, but still indicated a tendency of lower pain scores with QLB (MD: −0.53, 95% CI: −1.08, 0.01 *I*^2^ = 88% *p* = 0.05) (Supplementary Figure 4). Similarly, results of the 48 h pain scores turned non-significant on sequential exclusion of the study by He et al. (21) (MD: −0.46, 95% CI: −1.21, 0.29 *I*^2^ = 72% *p* = 0.23) (Supplementary Figure 5) and He et al. (30) (MD: −0.69, 95% CI: −1.88, 0.50 *I*^2^ = 89% *p* = 0.26) (Supplementary Figure 6). There was no change in the significance of remaining pain scores on exclusion of any study.

**Figure 6:**
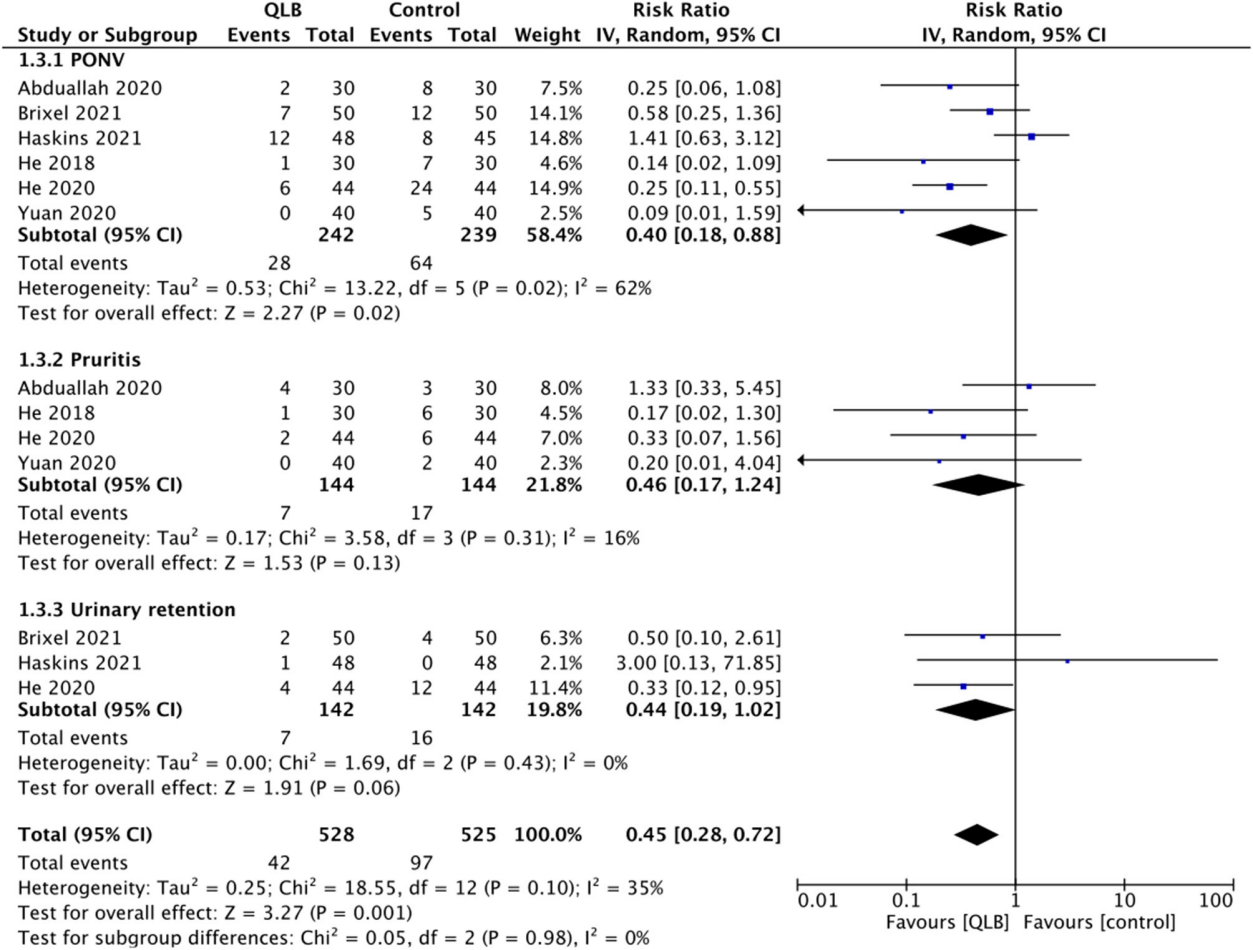
Meta-analysis of adverse events between QLB and control groups.

Adverse events were reported only by RCTs, and the common adverse events noted were post-operative nausea and vomiting (PONV), pruritis, and urinary retention. On pooled analysis, we noted a statistically significant reduced risk of PONV (RR: 0.40, 95% CI: 0.18, 0.88 *I*^2^ = 62% *p* = 0.02) in patients receiving QLB but no difference in the risk of pruritis (RR: 0.46, 95% CI: 0.17, 1.24 *I*^2^ = 16% *p* = 0.13) and urinary retention (RR: 0.44, 95% CI: 0.19, 1.02 *I*^2^ = 0% *p* = 0.06) (Figure 6).

### Certainty of Evidence

Assessment of certainty of evidence based on GRADE is presented in Supplementary Table 3. GRADE assessment was carried out only for the outcomes from RCTs and not from retrospective studies. Overall, the certainty of the evidence was “moderate” for total analgesic consumption and pain scores and “low-moderate” for complications.

### Meta-Regression Analysis

Results of meta-regression analysis for the outcomes of 24-h total analgesic consumption, 12 and 24 h pain scores are presented in Supplementary Tables 4–6, respectively. Scatter plots of the meta-regression analysis are presented as Supplementary Figures 7–21. None of the included covariates were found to significantly affect 24-h total analgesic consumption or 12 h pain scores. For the outcome of 24 h pain scores, only type of local anesthetic was found to significantly impact pain scores. Use of bupivacaine vs. ropivacaine was found to reduce mean difference indicating better pain control with ropivacaine (*p* = 0.02).

### Descriptive Analysis

Three studies were not included in the meta-analysis. The retrospective study of McCrum et al. (23) compared outcomes of 28 patients with QLB with 28 patients receiving no block. The authors noted a statistically significant reduction of opioid consumption in the entire post-operative period for patients receiving QLB as compared to controls (QLB: 6.53 ± 7.61 vs. No block: 14.02 ± 0.38, *p* < 0.001). Pain at discharge was also significantly lower in the QLB group (2.57 ± 2.29) as compared to the control group (4.18 ± 2.14) (*p* = 0.015). In the second study, Stuart Green et al. (22) retrospectively compared outcomes of 10 patients receiving QLB with 10 patients not receiving any block. The authors noted no statistically significant difference in fentanyl utilization in the post-anesthetic care unit (PACU) (QLB: 45 ± 59.8 vs. control: 25 ± 42.4; *p* = 0.4) or in the 24 h mean VAS scores (QLB: 5.48 ± 2 vs. control: 6.45 ± 2.5; *p* = 0.38) between the two groups. The RCT of Wilson et al. (32) compared outcomes of 22 patients receiving QLB with 24 controls. The authors noted significantly reduced opioid consumption in the PACU in patients receiving QLB as compared to controls [mean (95% CI); QLB: 8.1 (6, 10.2) vs. control 11.3 (9, 13.6) (*p* = 0.03)]. Pain scores at PACU discharge were, however, not significantly different between the two groups (57.9 ± 22.2 vs. 59.2 ± 22.6) (*p* = 0.84).

## Discussion

The current systematic review and meta-analysis aimed to summarize evidence on the efficacy of QLB for patients undergoing hip surgeries. Our analysis demonstrates that hip surgery patients receiving QLB have significantly reduced total opioid consumption in the first 24 h and have reduced pain scores up to 48 h after the procedure. Furthermore, the incidence of PONV is reduced in patients receiving QLB.

An essential element of post-operative care of hip surgery patients involves optimal pain control, reduced opioid consumption, and early mobilization. Indeed, the enhanced recovery after surgery protocol, which includes multimodal analgesia and early mobilization, has been shown to reduce the length of hospital stay and incidence of complications after hip surgeries (34). While epidural anesthesia and nerve blocks are popular methods of pain control, they can also delay ambulation which can slow down post-operative recovery (31). Therefore, there is a need for less invasive regional anesthetic techniques like the interfacial plane blocks (35). Unlike peripheral regional blocks which have specific neural endpoints, interfacial nerve blocks like the QLB are injected in tissue planes and target variable nerve endings depending upon the spread of local anesthetic (35). Over the past decade, the QLB has been used for proving analgesia after several surgical procedures, but its efficacy for hip surgeries is still unclear (14, 15).

In our analysis, we noted a statistically significant reduction of opioid consumption in the first 24-h after surgery in patients receiving the QLB. While the results were derived mostly from RCTs, one included retrospective study also demonstrated similar conclusions. Qualitative analysis of studies revealed similar outcomes for two of the three studies. Our results are in agreement with prior meta-analysis studies of Jin et al. (14) and Korgvee et al. (15) who have also reported significantly reduced 24-h opioid consumption with QLB on a pooled analysis of studies mostly on cesarean sections, renal or abdominal surgeries. However, high heterogeneity in the quantitative analysis (98%) downgraded the certainty of evidence in our study to “moderate.” This heterogeneity was persistent even on sub-group analysis based on study type, and after excluding two studies on arthroscopy. Similar high heterogeneity has been noted in the previous meta-analysis of the QLB (15). We believe that it stems from the different multimodal analgesia protocols used in the included studies, leading to significantly variable opioid consumption amongst the included patients. This is further strengthened by the fact that none of the included covariates in the meta-regression analysis were found to impact outcomes.

In the second part of our analysis, we noted that patients receiving QLB had consistently lower pain scores at all time points from 2–4 to 48 h. The results remained statistically significant even on the exclusion of the retrospective studies, thereby demonstrating the stability of our results. Nevertheless, the crucial detail lies in the MD between the two groups at the different time intervals. In the analysis of all included studies, the MD in pain scores between QLB and control ranged from −0.57 to −1.45, while in the analysis of RCTs, it ranged from −0.60 to −1.45. Considering these values with the concept of “minimally important clinical difference” (MCID) for pain scores, the pain reduction may not have been clinically relevant (36). In a recent study, Laigaard et al. (37) have shown that MCID for pain at rest after hip/knee arthroplasty was 1.5 and MCID for 24-h opioid consumption was 10 mg in morphine equivalents. Given the small MD in pain scores and MD of 7.65 for total opioid consumption, detected in our analysis, we believe that the clinical relevance of analgesia offered by QLB in hip surgeries may be questionable, despite the statistically significant results. Due to the high heterogeneity amongst the studies, further trials are required to elucidate if the use of QLB in hip surgeries leads to clinically important pain reduction. Secondly, it is important to note that two different local anesthetics were used by the included studies, namely, bupivacaine and ropivacaine. Meta-regression analysis based on type of drug did not demonstrate significant results for 12 h pain scores but indicated that ropivacaine may offer better pain reduction at 24 h. However, the results should be interpreted with caution due to the limited number of studies in the analysis.

An important limitation of the review is that we could not separate outcomes of different QLB *via* a subgroup analysis due to limited data. Only a meta-regression of important variables was performed which indicated no effect of type of QLB on analgesic consumption and 12, 24 h pain scores. Research has suggested that the approach of the QLB (anterior, lateral, or posterior) can result in variable anesthetic spread ranging from T6 to L4 (13). However, the systematic review of Jin et al. (14) has reported that the approach of QLB does not seem to impact the analgesic efficacy. While most of the studies included in our review utilized the posterior QLB, a few did report the use of anterior or lateral QLB but with variable results. While Haskins et al. (33) reported the lack of beneficial effect with anterior QLB in hip arthroscopic surgeries, Kukreja et al. (26) found a statistically significant reduction in pain scores and opioid consumption with anterior QLB in patients undergoing hip arthroplasties. Another retrospective study of Kinjo et al. (27) also reported no significant difference in pain scores with anterior QLB. Such variability of results can be partly explained by the lack of clarity on the mechanism of action of interfacial blocks (31). Even cadaveric studies investigating dye spread following QLB have produced inconsistent results, with one study (38) reporting dye spread up to the lumbar nerve roots with anterior QLB while another reporting no such effect (39). It has also been postulated that small changes in the needle position and different approaches of QLB can significantly alter the quality of analgesia offered by the block (27). On the other hand, Brixel et al. (31) have demonstrated that even with rigorous control of needle position in the posterior QLB, the anesthetic solution had an unpredictable spread. The authors have suggested that the spread of the anesthetic solution after QLB depends more on tissue compliance rather than the needle position in the thoracolumbar interfascial plane. Since interfacial nerve blocks are relatively new in the field of regional anesthesia, with limited studies on their exact mechanism, further research is needed to shed light on the variability of analgesia with different approaches of these blocks.

Our meta-analysis demonstrated a significantly reduced risk of PONV with QLB. This provides indirect evidence of reduced opioid usage in the QLB group with subsequent reduction of opioid-related side effects. However, there were no differences in the risk of other opioid-related adverse events like pruritis or urinary continence probably due to the limited number of studies in the meta-analysis. Important block-related adverse events of clinical relevance like muscle weakness were not universally reported by the included studies and hence could not be analyzed.

Our results concur with a recently published meta-analysis of Koo et al. (40) which has also assessed the efficacy of QLB for hip surgeries. The authors in their pooled analysis of nine RCTs have demonstrated reduced post-operative opioid consumption and incidence of PONV with the QLB in patients undergoing hip surgeries. However, unlike their review, our study also included non-RCTs to present comprehensive evidence on the efficacy of QLB for hip surgeries. Furthermore, we also conducted subgroup analysis based on the study type and presented separate data for arthroplasty studies in our review.

The results of our review should be interpreted with the following limitations. Firstly, as mentioned earlier, there was significant heterogeneity in our analysis which can be attributed to the methodological differences in the included studies. Other than differences in the post-operative analgesic regimen, the studies varied in the type of surgery, type and volume of local anesthetics, the use of adjuvants, the timing of QLB, and the approach of the block. We were unable to perform subgroup analysis for these variables due to limited data. Therefore, our meta-analysis was unable to judge which is the best approach for QLB for pain control in hip surgeries. Secondly, only 13 studies were available for inclusion in the review, of which just nine were RCTs. Variability in reporting of data further reduced the number of studies available for the meta-analysis. Thirdly, important outcomes like time to first analgesic request and muscle weakness associated with the block, the ideal volume of anesthetic required for the block, and the level of sensory anesthesia provided by the block could not be analyzed due to the lack of reporting from the included studies. Lastly, several different practitioners with variable experience were involved in administering the QLB in the included studies. The impact of practitioner skill on the study outcomes could not be assessed.

## Clinical Significance

The treatment strategy for patients undergoing hip surgery should be focused at providing optimal analgesia and improved patient satisfaction in the immediate post-operative period. In this context, we believe that the QLB can be a valuable regional anesthetic technique for patients undergoing hip surgeries. We recommend that anesthetists should routinely use the QLB along with their standard analgesic regimen to provide better pain control in these patients. Use of only systemic analgesics may not be recommended since the addition of QLB seems to be safe without any serious adverse events. Future research focusing on the efficacy of QLB on specific types of hip surgeries like arthroscopy and arthroplasty shall enhance our understanding of the efficacy of this block for pain control. Also, further research is also needed to analyze which is the best QLB approach for patients undergoing hip surgery.

## Conclusions

Our systematic review and meta-analysis indicated that the QLB as a part of multimodal analgesia protocol reduces opioid consumption and pain scores in patients undergoing hip surgeries. The certainty of evidence based on GRADE was moderate. Despite the statistically significant results, the clinical relevance of the analgesic efficacy offered by the QLB is debatable owing to the small effect size. Further studies assessing the efficacy of QLB against standard analgesic protocols are needed to strengthen the current evidence, and should also compare outcomes with different approaches of QLB to provide evidence on what constitutes the best approach.

## Data Availability Statement

The original contributions presented in the study are included in the article/Supplementary Material, further inquiries can be directed to the corresponding authors.

## Author Contributions

JLi and CW conceptualized and designed the study. JH and YL did literature search and data collection. HL and JLiu analyzed the data. JLi wrote the paper. JLiu and CJ reviewed and edited the manuscript. All authors read and approved the final manuscript.

## Funding

This work was funded by Science and Technology Research Project of Guangdong Provincial Hospital of Chinese Medicine (No. YN2019ML08), Science and Technology Program of Guangzhou (202102010273), Science and Technology Planning Project of Guangdong Province (No. 2020A1414050050), the Project of Guangdong Provincial Department of Finance [Nos. (2014)157, (2018)8], and National key research and development program (2021YFC1712804).

## Conflict of Interest

The authors declare that the research was conducted in the absence of any commercial or financial relationships that could be construed as a potential conflict of interest.

## Publisher's Note

All claims expressed in this article are solely those of the authors and do not necessarily represent those of their affiliated organizations, or those of the publisher, the editors and the reviewers. Any product that may be evaluated in this article, or claim that may be made by its manufacturer, is not guaranteed or endorsed by the publisher.

## Supplementary Material

Supplementary Figure 1 Funnel plot for the meta-analysis of 24-h total analgesic consumption between QLB and control groups.

Supplementary Figure 2 Meta-analysis of 24-h total analgesic consumption between QLB and control groups with exclusion of studies on arthroscopy.

Supplementary Figure 3 Meta-analysis of pain scores between QLB and control groups with the exclusion of retrospective studies.

Supplementary Figure 4 Meta-analysis of pain scores between QLB and control groups with exclusion of the study of Yuan et al. (24 h subgroup).

Supplementary Figure 5 Meta-analysis of pain scores between QLB and control groups with exclusion of the study of He et al. (21) (48 h subgroup).

Supplementary Figure 6 Meta-analysis of pain scores between QLB and control groups with exclusion of the study of He et al. (30) (48 h subgroup).

Supplementary Figure 7 Meta-regression plot assessing the impact of mean age on 24-h total analgesic consumption.

Supplementary Figure 8 Meta-regression plot assessing the impact of male gender on 24-h total analgesic consumption.

Supplementary Figure 9 Meta-regression plot assessing the impact of sample size on 24-h total analgesic consumption.

Supplementary Figure 10 Meta-regression plot assessing the impact of QLB type on 24-h total analgesic consumption.

Supplementary Figure 11 Meta-regression plot assessing the impact of local anesthetic type on 24-h total analgesic consumption.

Supplementary Figure 12 Meta-regression plot assessing the impact of mean age on 12 h pain scores.

Supplementary Figure 13 Meta-regression plot assessing the impact of male gender on 12 h pain scores.

Supplementary Figure 14 Meta-regression plot assessing the impact of sample size on 12 h pain scores.

Supplementary Figure 15 Meta-regression plot assessing the impact of QLB type on 12 h pain scores.

Supplementary Figure 16 Meta-regression plot assessing the impact of local anesthetic on 12 h pain scores.

Supplementary Figure 17 Meta-regression plot assessing the impact of mean age on 24 h pain scores.

Supplementary Figure 18 Meta-regression plot assessing the impact of male gender on 24 h pain scores.

Supplementary Figure 19 Meta-regression plot assessing the impact of sample size on 24 h pain scores.

Supplementary Figure 20 Meta-regression plot assessing the impact of QLB type on 24 h pain scores.

Supplementary Figure 21 Meta-regression plot assessing the impact of local anesthetic on 24 h pain scores.

Supplementary Table 1 PRISMA checklist.

Supplementary Table 2 Search strategy.

Supplementary Table 3 GRADE assessment of evidence.

Supplementary Table 4 Meta-regression analysis for the heterogeneity of post-operative 24 h total analgesic consumption.

Supplementary Table 5 Meta-regression analysis for the heterogeneity of 12 h pain scores.

Supplementary Table 6 Meta-regression analysis for the heterogeneity of 24 h pain scores.
