# Supplementary material for: Efficacy of Quadratus Lumborum Block for Pain Control in Patients Undergoing Hip Surgeries: A Systematic Review and Meta-Analysis
Source: Front Med (Lausanne). 2022 Feb 3;8:771859. doi: 10.3389/fmed.2021.771859 (PMC8850973; doi:10.3389/fmed.2021.771859)

Supplementary figure 1

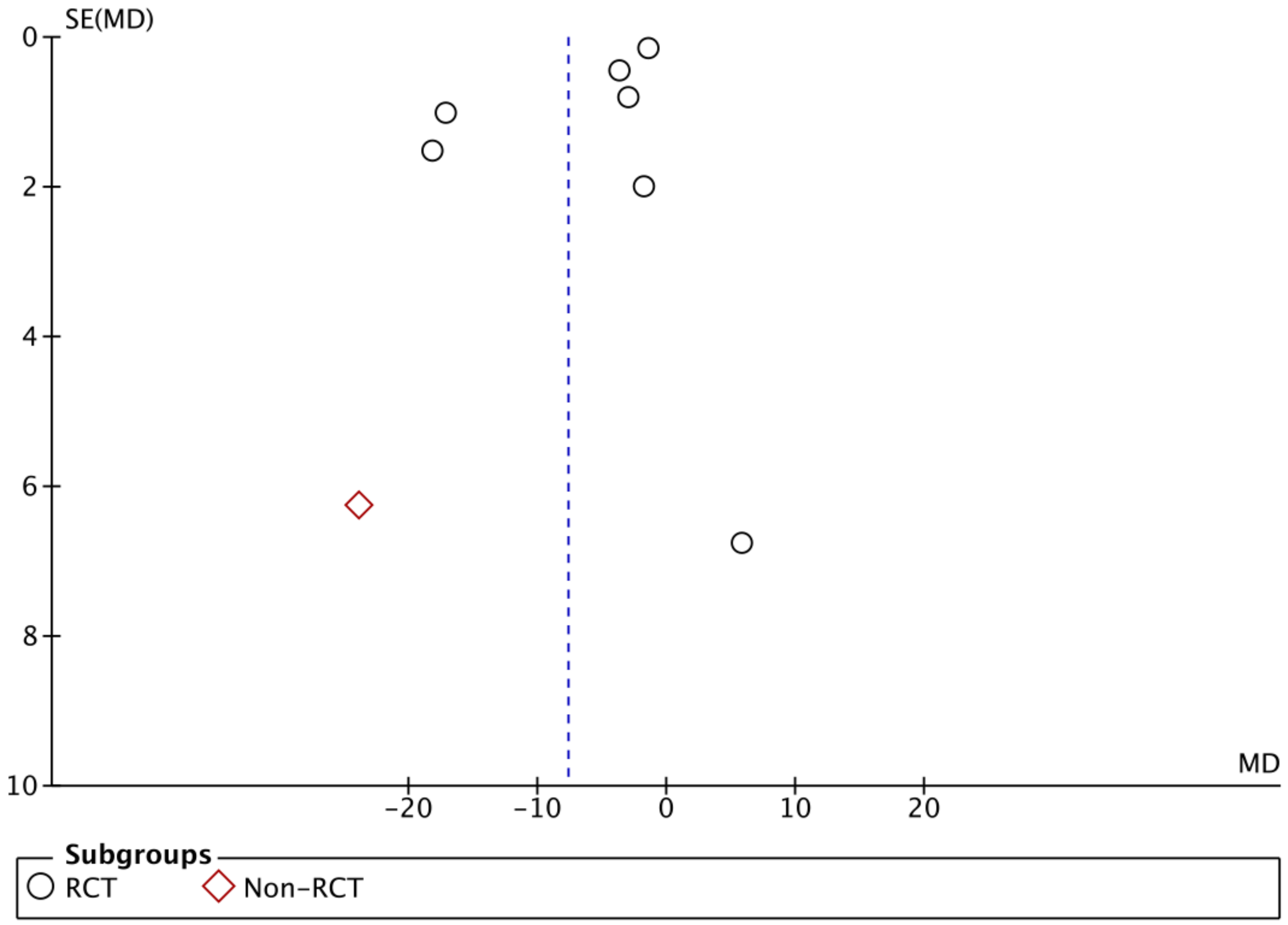

Supplementary figure 2

Study or Subgroup	QLB Mean	QLB SD	QLB Total	Control Mean	Control SD	Control Total	Weight	Mean Difference IV, Random, 95% CI
**1.1.1 RCT**								
Abduallah 2020	5.6	3.22	30	8.5	3.06	30	18.3%	−2.90 [−4.49, −1.31]
Brixel 2021	13.67	10.68	50	15.33	9.16	50	17.1%	−1.66 [−5.56, 2.24]
Haskins 2021	68.61	38.51	44	62.67	22.97	45	0.0%	5.94 [−7.27, 19.15]
He 2020	16	7.1	44	34.1	7.1	44	17.7%	−18.10 [−21.07, −15.13]
Kukreja1 2019	30.05	3.8	36	47.14	4.72	35	18.1%	−17.09 [−19.09, −15.09]
Tulgar 2018	5.48	1.52	20	9.04	1.44	20	18.4%	−3.56 [−4.48, −2.64]
Yuan 2020	24.8	0.19	40	26.15	1.08	40	0.0%	−1.35 [−1.69, −1.01]
**Subtotal (95% CI)**			**180**			**179**	**89.6%**	**−8.67 [−14.90, −2.44]**

Heterogeneity: $Tau^2 = 48.95$; $Chi^2 = 228.83$, df = 4 (P < 0.00001); $I^2 = 98\%$
Test for overall effect: Z = 2.73 (P = 0.006)

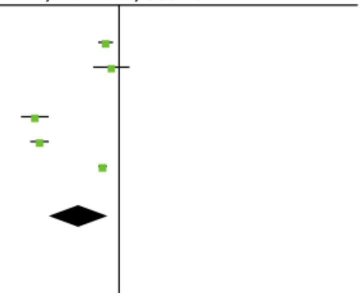

Supplementary figure 3

Study or Subgroup	QLB Mean	QLB SD	QLB Total	Control Mean	Control SD	Control Total	Weight	Mean Difference IV, Random, 95% CI
**1.2.1 2–4 hours**								
Abduallah 2020	2.33	2.33	30	3.33	3.89	30	5.5%	−1.00 [−2.62, 0.62]
Brixel 2021	2.47	1.58	50	2.61	2.35	50	14.7%	−0.14 [−0.92, 0.64]
Haskins 2021	4.1	2.1	48	4.6	2.4	46	12.4%	−0.50 [−1.41, 0.41]
He 2020	0.85	0.53	44	1.04	0.86	44	25.8%	−0.19 [−0.49, 0.11]
Tulgar 2018	1	0.65	20	2	0.46	20	24.7%	−1.00 [−1.35, −0.65]
Yuan 2020	3	1.54	40	4	1.54	40	16.9%	−1.00 [−1.67, −0.33]
**Subtotal (95% CI)**			**232**			**230**	**100.0%**	**−0.60 [−1.02, −0.18]**
Heterogeneity: $Tau^2 = 0.16$; $Chi^2 = 15.04$, df = 5 (P = 0.01); $I^2 = 67\%$								
Test for overall effect: Z = 2.80 (P = 0.005)								
**1.2.2 6–8 hours**								
Abduallah 2020	4.33	3.89	30	5	3.11	30	8.1%	−0.67 [−2.45, 1.11]
Brixel 2021	1.13	1.6	50	1.75	2.3	50	16.3%	−0.62 [−1.40, 0.16]
He 2018	1	0.3	30	3.5	0.9	30	20.3%	−2.50 [−2.84, −2.16]
He 2020	1.32	0.72	44	2.74	1.04	44	20.0%	−1.42 [−1.79, −1.05]
Tulgar 2018	1.3	0.57	20	2.35	1.27	20	18.0%	−1.05 [−1.66, −0.44]
Yuan 2020	2	1.54	40	3.83	1.53	40	17.4%	−1.83 [−2.50, −1.16]
**Subtotal (95% CI)**			**214**			**214**	**100.0%**	**−1.45 [−2.09, −0.81]**
Heterogeneity: $Tau^2 = 0.50$; $Chi^2 = 36.39$, df = 5 (P < 0.00001); $I^2 = 86\%$								
Test for overall effect: Z = 4.45 (P < 0.00001)								
**1.2.3 12 hours**								
Abduallah 2020	2.67	0.78	30	3	1.56	30	14.7%	−0.33 [−0.95, 0.29]
Brixel 2021	0.76	0.79	50	1.19	1.53	50	15.2%	−0.43 [−0.91, 0.05]
He 2018	1.2	0.5	30	3.8	0.9	30	15.5%	−2.60 [−2.97, −2.23]
He 2020	2.51	0.95	44	3.81	0.77	44	15.5%	−1.30 [−1.66, −0.94]
Kukreja1 2019	2.34	2.76	36	3.33	3.31	35	11.1%	−0.99 [−2.41, 0.43]
Tulgar 2018	2.35	0.99	20	2.15	0.59	20	15.1%	0.20 [−0.31, 0.71]
Yuan 2020	1	1.54	40	3	3.07	40	12.8%	−2.00 [−3.06, −0.94]
**Subtotal (95% CI)**			**250**			**249**	**100.0%**	**−1.05 [−1.91, −0.20]**
Heterogeneity: $Tau^2 = 1.19$; $Chi^2 = 104.95$, df = 6 (P < 0.00001); $I^2 = 94\%$								
Test for overall effect: Z = 2.42 (P = 0.02)								
**1.2.4 24 hours**								
Abduallah 2020	1.33	2.34	30	1.33	2.34	30	9.7%	0.00 [−1.18, 1.18]
Brixel 2021	0.85	1.53	50	1.19	1.53	50	13.5%	−0.34 [−0.94, 0.26]
Haskins 2021	3.3	2.5	46	3.8	2.1	46	11.3%	−0.50 [−1.44, 0.44]
He 2018	2	0.5	30	3.3	1.1	30	14.4%	−1.30 [−1.73, −0.87]
He 2020	2.39	0.83	44	3.38	0.96	44	14.7%	−0.99 [−1.36, −0.62]
Kukreja1 2019	2.31	2.22	36	4.06	2.48	35	10.3%	−1.75 [−2.85, −0.65]
Tulgar 2018	2.1	0.45	20	1.95	0.39	29	15.2%	0.15 [−0.09, 0.39]
Yuan 2020	0.67	0.77	40	3	3.07	40	11.0%	−2.33 [−3.31, −1.35]
**Subtotal (95% CI)**			**296**			**304**	**100.0%**	**−0.85 [−1.45, −0.25]**
Heterogeneity: $Tau^2 = 0.61$; $Chi^2 = 66.40$, df = 7 (P < 0.00001); $I^2 = 89\%$								
Test for overall effect: Z = 2.76 (P = 0.006)								
**1.2.5 48 hours**								
He 2018	1.6	0.5	30	3	0.7	30	44.4%	−1.40 [−1.71, −1.09]
He 2020	1.56	0.5	44	2.38	0.73	44	46.5%	−0.82 [−1.08, −0.56]
Kukreja1 2019	3.33	2.58	36	4.11	3.78	35	9.1%	−0.78 [−2.29, 0.73]
**Subtotal (95% CI)**			**110**			**109**	**100.0%**	**−1.07 [−1.57, −0.57]**
Heterogeneity: $Tau^2 = 0.12$; $Chi^2 = 8.06$, df = 2 (P = 0.02); $I^2 = 75\%$								
Test for overall effect: Z = 4.21 (P < 0.0001)								

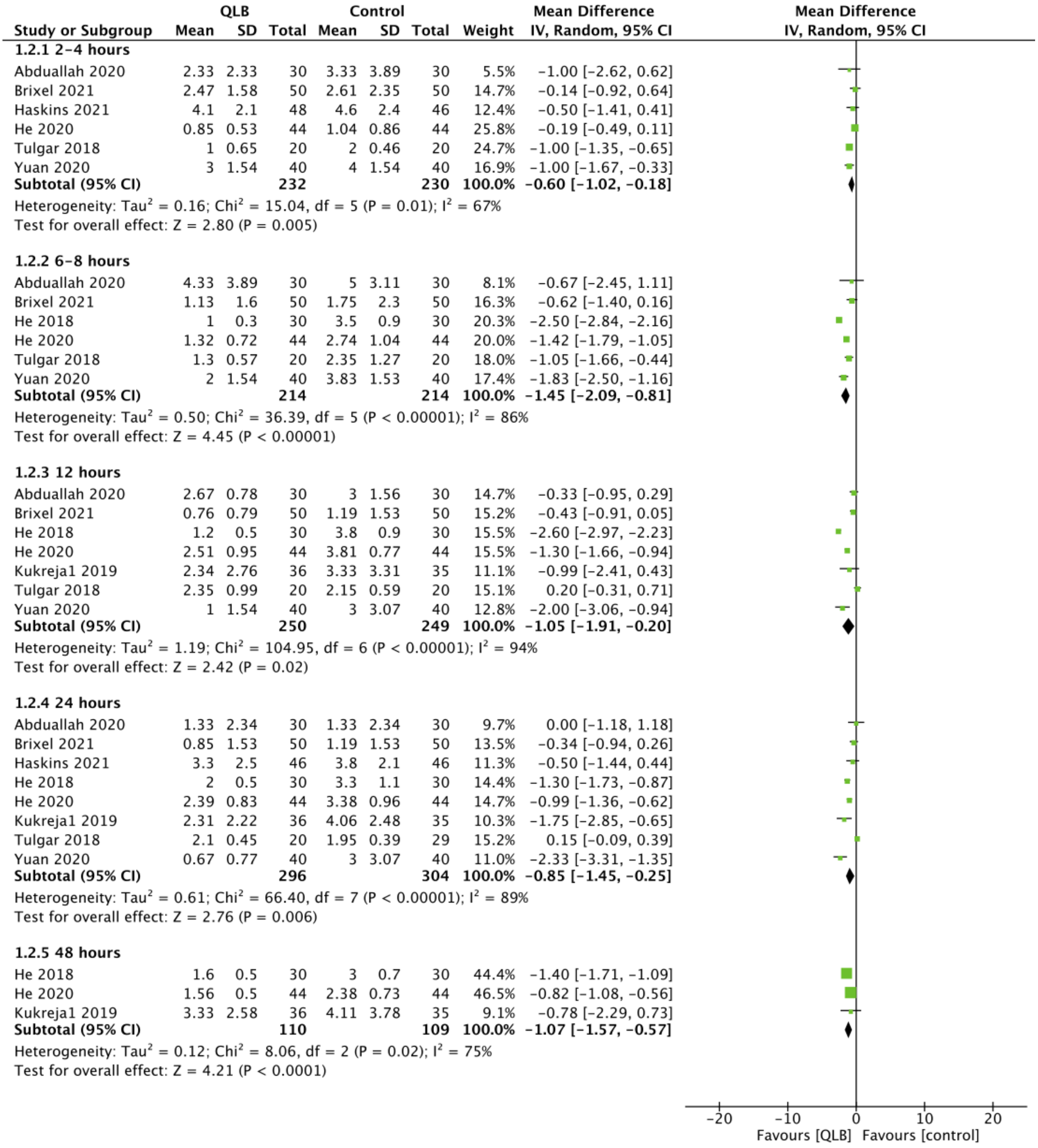

Supplementary figure 4

Study or Subgroup	QLB Mean	QLB SD	QLB Total	Control Mean	Control SD	Control Total	Weight	Mean Difference IV, Random, 95% CI
**1.2.1 2-4 hours**								
Abduallah 2020	2.33	2.33	30	3.33	3.89	30	5.1%	−1.00 [−2.62, 0.62]
Brixel 2021	2.47	1.58	50	2.61	2.35	50	13.9%	−0.14 [−0.92, 0.64]
Haskins 2021	4.1	2.1	48	4.6	2.4	46	11.7%	−0.50 [−1.41, 0.41]
He 2020	0.85	0.53	44	1.04	0.86	44	25.1%	−0.19 [−0.49, 0.11]
Kinjo 2019	4.5	3.5	15	4.4	1.5	54	4.2%	0.10 [−1.72, 1.92]
Tulgar 2018	1	0.65	20	2	0.46	20	23.9%	−1.00 [−1.35, −0.65]
Yuan 2020	3	1.54	40	4	1.54	40	16.1%	−1.00 [−1.67, −0.33]
**Subtotal (95% CI)**			**247**			**284**	**100.0%**	**−0.57 [−0.98, −0.17]**
Heterogeneity: $Tau^2 = 0.15$; $Chi^2 = 15.53$, df = 6 (P = 0.02); $I^2 = 61\%$								
Test for overall effect: Z = 2.78 (P = 0.005)								
**1.2.2 6-8 hours**								
Abduallah 2020	4.33	3.89	30	5	3.11	30	8.1%	−0.67 [−2.45, 1.11]
Brixel 2021	1.13	1.6	50	1.75	2.3	50	16.3%	−0.62 [−1.40, 0.16]
He 2018	1	0.3	30	3.5	0.9	30	20.3%	−2.50 [−2.84, −2.16]
He 2020	1.32	0.72	44	2.74	1.04	44	20.0%	−1.42 [−1.79, −1.05]
Tulgar 2018	1.3	0.57	20	2.35	1.27	20	18.0%	−1.05 [−1.66, −0.44]
Yuan 2020	2	1.54	40	3.83	1.53	40	17.4%	−1.83 [−2.50, −1.16]
**Subtotal (95% CI)**			**214**			**214**	**100.0%**	**−1.45 [−2.09, −0.81]**
Heterogeneity: $Tau^2 = 0.50$; $Chi^2 = 36.39$, df = 5 (P < 0.00001); $I^2 = 86\%$								
Test for overall effect: Z = 4.45 (P < 0.00001)								
**1.2.3 12 hours**								
Abduallah 2020	2.67	0.78	30	3	1.56	30	13.0%	−0.33 [−0.95, 0.29]
Brixel 2021	0.76	0.79	50	1.19	1.53	50	13.4%	−0.43 [−0.91, 0.05]
He 2018	1.2	0.5	30	3.8	0.9	30	13.7%	−2.60 [−2.97, −2.23]
He 2020	2.51	0.95	44	3.81	0.77	44	13.7%	−1.30 [−1.66, −0.94]
Kukreja 2019	2.54	2.88	79	4.12	3.98	159	12.0%	−1.58 [−2.47, −0.69]
Kukreja1 2019	2.34	2.76	36	3.33	3.31	35	9.6%	−0.99 [−2.41, 0.43]
Tulgar 2018	2.35	0.99	20	2.15	0.59	20	13.4%	0.20 [−0.31, 0.71]
Yuan 2020	1	1.54	40	3	3.07	40	11.2%	−2.00 [−3.06, −0.94]
**Subtotal (95% CI)**			**329**			**408**	**100.0%**	**−1.12 [−1.89, −0.34]**
Heterogeneity: $Tau^2 = 1.11$; $Chi^2 = 105.55$, df = 7 (P < 0.00001); $I^2 = 93\%$								
Test for overall effect: Z = 2.82 (P = 0.005)								
**1.2.4 24 hours**								
Abduallah 2020	1.33	2.34	30	1.33	2.34	30	9.0%	0.00 [−1.18, 1.18]
Brixel 2021	0.85	1.53	50	1.19	1.53	50	13.3%	−0.34 [−0.94, 0.26]
Haskins 2021	3.3	2.5	46	3.8	2.1	46	10.7%	−0.50 [−1.44, 0.44]
He 2018	2	0.5	30	3.3	1.1	30	14.4%	−1.30 [−1.73, −0.87]
He 2020	2.39	0.83	44	3.38	0.96	44	14.8%	−0.99 [−1.36, −0.62]
Kukreja 2019	4.56	2.47	79	4.22	2.63	159	12.7%	0.34 [−0.34, 1.02]
Kukreja1 2019	2.31	2.22	36	4.06	2.48	35	9.6%	−1.75 [−2.85, −0.65]
Tulgar 2018	2.1	0.45	20	1.95	0.39	29	15.4%	0.15 [−0.09, 0.39]
Yuan 2020	0.67	0.77	40	3	3.07	40	0.0%	−2.33 [−3.31, −1.35]
**Subtotal (95% CI)**			**335**			**423**	**100.0%**	**−0.53 [−1.08, 0.01]**
Heterogeneity: $Tau^2 = 0.48$; $Chi^2 = 56.59$, df = 7 (P < 0.00001); $I^2 = 88\%$								
Test for overall effect: Z = 1.93 (P = 0.05)								
**1.2.5 48 hours**								
He 2018	1.6	0.5	30	3	0.7	30	31.9%	−1.40 [−1.71, −1.09]
He 2020	1.56	0.5	44	2.38	0.73	44	32.7%	−0.82 [−1.08, −0.56]
Kukreja 2019	4.11	2.4	79	3.95	2.63	159	24.4%	0.16 [−0.51, 0.83]
Kukreja1 2019	3.33	2.58	36	4.11	3.78	35	11.0%	−0.78 [−2.29, 0.73]
**Subtotal (95% CI)**			**189**			**268**	**100.0%**	**−0.76 [−1.37, −0.16]**
Heterogeneity: $Tau^2 = 0.27$; $Chi^2 = 19.78$, df = 3 (P = 0.0002); $I^2 = 85\%$								
Test for overall effect: Z = 2.47 (P = 0.01)								

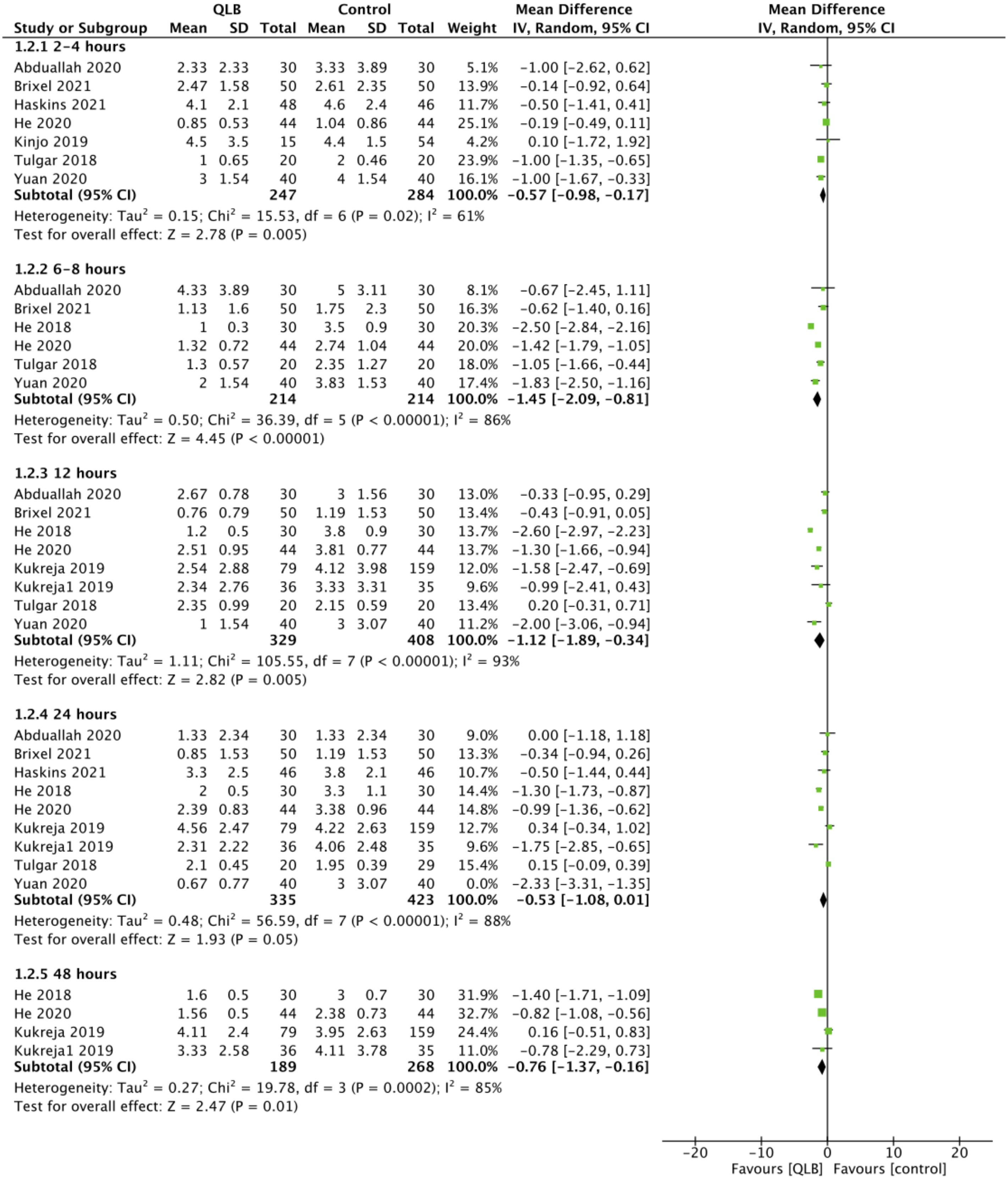

Supplementary figure 5

Study or Subgroup	QLB Mean	QLB SD	QLB Total	Control Mean	Control SD	Control Total	Weight	Mean Difference IV, Random, 95% CI
**1.2.1 2-4 hours**								
Abduallah 2020	2.33	2.33	30	3.33	3.89	30	5.1%	-1.00 [-2.62, 0.62]
Brixel 2021	2.47	1.58	50	2.61	2.35	50	13.9%	-0.14 [-0.92, 0.64]
Haskins 2021	4.1	2.1	48	4.6	2.4	46	11.7%	-0.50 [-1.41, 0.41]
He 2020	0.85	0.53	44	1.04	0.86	44	25.1%	-0.19 [-0.49, 0.11]
Kinjo 2019	4.5	3.5	15	4.4	1.5	54	4.2%	0.10 [-1.72, 1.92]
Tulgar 2018	1	0.65	20	2	0.46	20	23.9%	-1.00 [-1.35, -0.65]
Yuan 2020	3	1.54	40	4	1.54	40	16.1%	-1.00 [-1.67, -0.33]
**Subtotal (95% CI)**			**247**			**284**	**100.0%**	**-0.57 [-0.98, -0.17]**
Heterogeneity: $Tau^2 = 0.15$; $Chi^2 = 15.53$, df = 6 (P = 0.02); $I^2 = 61\%$								
Test for overall effect: Z = 2.78 (P = 0.005)								
**1.2.2 6-8 hours**								
Abduallah 2020	4.33	3.89	30	5	3.11	30	8.1%	-0.67 [-2.45, 1.11]
Brixel 2021	1.13	1.6	50	1.75	2.3	50	16.3%	-0.62 [-1.40, 0.16]
He 2018	1	0.3	30	3.5	0.9	30	20.3%	-2.50 [-2.84, -2.16]
He 2020	1.32	0.72	44	2.74	1.04	44	20.0%	-1.42 [-1.79, -1.05]
Tulgar 2018	1.3	0.57	20	2.35	1.27	20	18.0%	-1.05 [-1.66, -0.44]
Yuan 2020	2	1.54	40	3.83	1.53	40	17.4%	-1.83 [-2.50, -1.16]
**Subtotal (95% CI)**			**214**			**214**	**100.0%**	**-1.45 [-2.09, -0.81]**
Heterogeneity: $Tau^2 = 0.50$; $Chi^2 = 36.39$, df = 5 (P < 0.00001); $I^2 = 86\%$								
Test for overall effect: Z = 4.45 (P < 0.00001)								
**1.2.3 12 hours**								
Abduallah 2020	2.67	0.78	30	3	1.56	30	13.0%	-0.33 [-0.95, 0.29]
Brixel 2021	0.76	0.79	50	1.19	1.53	50	13.4%	-0.43 [-0.91, 0.05]
He 2018	1.2	0.5	30	3.8	0.9	30	13.7%	-2.60 [-2.97, -2.23]
He 2020	2.51	0.95	44	3.81	0.77	44	13.7%	-1.30 [-1.66, -0.94]
Kukreja 2019	2.54	2.88	79	4.12	3.98	159	12.0%	-1.58 [-2.47, -0.69]
Kukreja1 2019	2.34	2.76	36	3.33	3.31	35	9.6%	-0.99 [-2.41, 0.43]
Tulgar 2018	2.35	0.99	20	2.15	0.59	20	13.4%	0.20 [-0.31, 0.71]
Yuan 2020	1	1.54	40	3	3.07	40	11.2%	-2.00 [-3.06, -0.94]
**Subtotal (95% CI)**			**329**			**408**	**100.0%**	**-1.12 [-1.89, -0.34]**
Heterogeneity: $Tau^2 = 1.11$; $Chi^2 = 105.55$, df = 7 (P < 0.00001); $I^2 = 93\%$								
Test for overall effect: Z = 2.82 (P = 0.005)								
**1.2.4 24 hours**								
Abduallah 2020	1.33	2.34	30	1.33	2.34	30	8.6%	0.00 [-1.18, 1.18]
Brixel 2021	0.85	1.53	50	1.19	1.53	50	11.9%	-0.34 [-0.94, 0.26]
Haskins 2021	3.3	2.5	46	3.8	2.1	46	9.9%	-0.50 [-1.44, 0.44]
He 2018	2	0.5	30	3.3	1.1	30	12.8%	-1.30 [-1.73, -0.87]
He 2020	2.39	0.83	44	3.38	0.96	44	13.0%	-0.99 [-1.36, -0.62]
Kukreja 2019	4.56	2.47	79	4.22	2.63	159	11.5%	0.34 [-0.34, 1.02]
Kukreja1 2019	2.31	2.22	36	4.06	2.48	35	9.0%	-1.75 [-2.85, -0.65]
Tulgar 2018	2.1	0.45	20	1.95	0.39	29	13.5%	0.15 [-0.09, 0.39]
Yuan 2020	0.67	0.77	40	3	3.07	40	9.7%	-2.33 [-3.31, -1.35]
**Subtotal (95% CI)**			**375**			**463**	**100.0%**	**-0.71 [-1.27, -0.15]**
Heterogeneity: $Tau^2 = 0.59$; $Chi^2 = 71.51$, df = 8 (P < 0.00001); $I^2 = 89\%$								
Test for overall effect: Z = 2.49 (P = 0.01)								
**1.2.5 48 hours**								
He 2018	1.6	0.5	30	3	0.7	30	0.0%	-1.40 [-1.71, -1.09]
He 2020	1.56	0.5	44	2.38	0.73	44	47.5%	-0.82 [-1.08, -0.56]
Kukreja 2019	4.11	2.4	79	3.95	2.63	159	36.0%	0.16 [-0.51, 0.83]
Kukreja1 2019	3.33	2.58	36	4.11	3.78	35	16.6%	-0.78 [-2.29, 0.73]
**Subtotal (95% CI)**			**159**			**238**	**100.0%**	**-0.46 [-1.21, 0.29]**
Heterogeneity: $Tau^2 = 0.29$; $Chi^2 = 7.17$, df = 2 (P = 0.03); $I^2 = 72\%$								
Test for overall effect: Z = 1.21 (P = 0.23)								

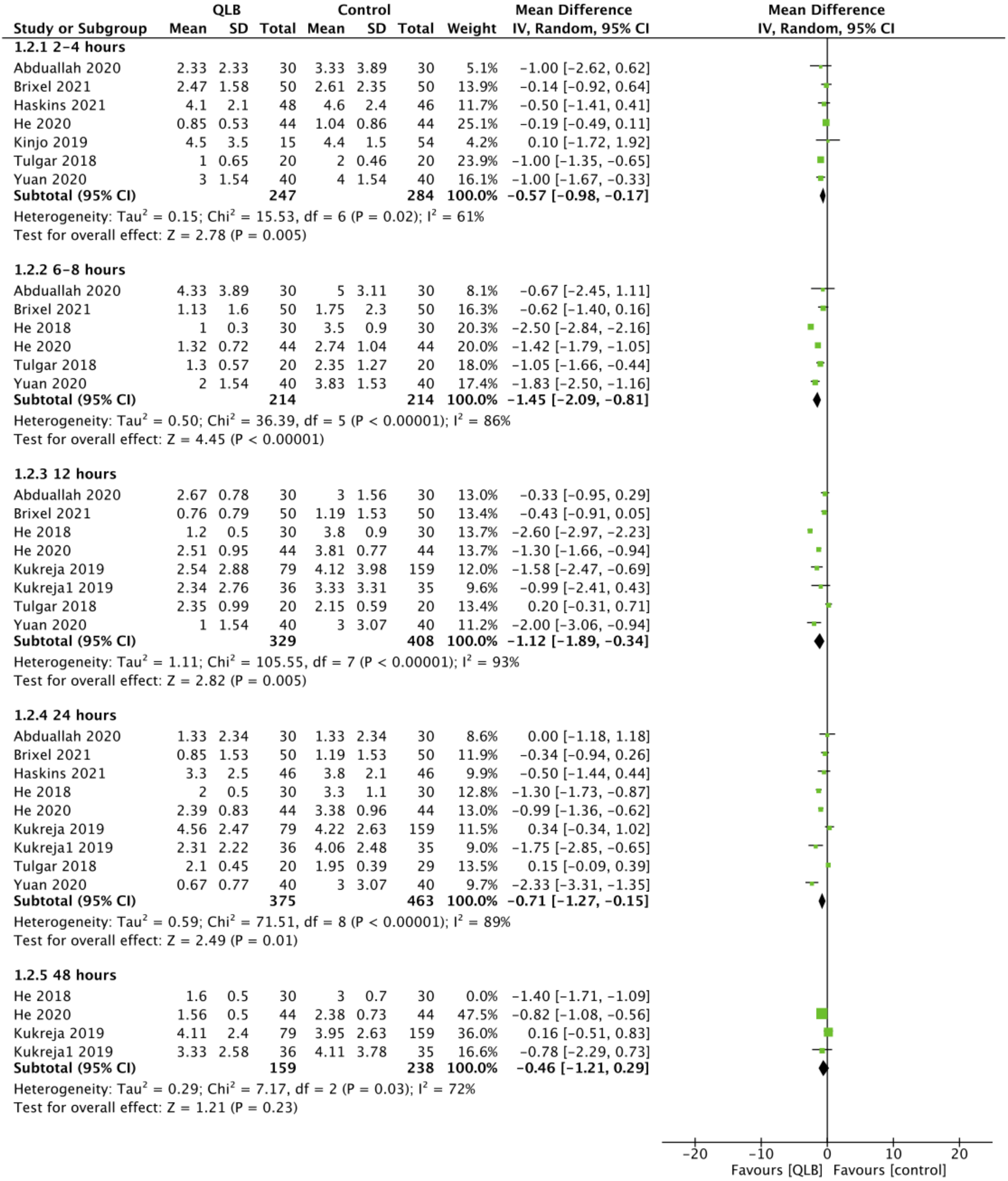

Supplementary figure 6

Study or Subgroup	QLB Mean	QLB SD	QLB Total	Control Mean	Control SD	Control Total	Weight	Mean Difference IV, Random, 95% CI
**1.2.1 2–4 hours**								
Abduallah 2020	2.33	2.33	30	3.33	3.89	30	5.1%	−1.00 [−2.62, 0.62]
Brixel 2021	2.47	1.58	50	2.61	2.35	50	13.9%	−0.14 [−0.92, 0.64]
Haskins 2021	4.1	2.1	48	4.6	2.4	46	11.7%	−0.50 [−1.41, 0.41]
He 2020	0.85	0.53	44	1.04	0.86	44	25.1%	−0.19 [−0.49, 0.11]
Kinjo 2019	4.5	3.5	15	4.4	1.5	54	4.2%	0.10 [−1.72, 1.92]
Tulgar 2018	1	0.65	20	2	0.46	20	23.9%	−1.00 [−1.35, −0.65]
Yuan 2020	3	1.54	40	4	1.54	40	16.1%	−1.00 [−1.67, −0.33]
**Subtotal (95% CI)**			**247**			**284**	**100.0%**	**−0.57 [−0.98, −0.17]**
Heterogeneity: $Tau^2 = 0.15$; $Chi^2 = 15.53$, df = 6 (P = 0.02); $I^2 = 61\%$								
Test for overall effect: Z = 2.78 (P = 0.005)								
**1.2.2 6–8 hours**								
Abduallah 2020	4.33	3.89	30	5	3.11	30	8.1%	−0.67 [−2.45, 1.11]
Brixel 2021	1.13	1.6	50	1.75	2.3	50	16.3%	−0.62 [−1.40, 0.16]
He 2018	1	0.3	30	3.5	0.9	30	20.3%	−2.50 [−2.84, −2.16]
He 2020	1.32	0.72	44	2.74	1.04	44	20.0%	−1.42 [−1.79, −1.05]
Tulgar 2018	1.3	0.57	20	2.35	1.27	20	18.0%	−1.05 [−1.66, −0.44]
Yuan 2020	2	1.54	40	3.83	1.53	40	17.4%	−1.83 [−2.50, −1.16]
**Subtotal (95% CI)**			**214**			**214**	**100.0%**	**−1.45 [−2.09, −0.81]**
Heterogeneity: $Tau^2 = 0.50$; $Chi^2 = 36.39$, df = 5 (P < 0.00001); $I^2 = 86\%$								
Test for overall effect: Z = 4.45 (P < 0.00001)								
**1.2.3 12 hours**								
Abduallah 2020	2.67	0.78	30	3	1.56	30	13.0%	−0.33 [−0.95, 0.29]
Brixel 2021	0.76	0.79	50	1.19	1.53	50	13.4%	−0.43 [−0.91, 0.05]
He 2018	1.2	0.5	30	3.8	0.9	30	13.7%	−2.60 [−2.97, −2.23]
He 2020	2.51	0.95	44	3.81	0.77	44	13.7%	−1.30 [−1.66, −0.94]
Kukreja 2019	2.54	2.88	79	4.12	3.98	159	12.0%	−1.58 [−2.47, −0.69]
Kukreja1 2019	2.34	2.76	36	3.33	3.31	35	9.6%	−0.99 [−2.41, 0.43]
Tulgar 2018	2.35	0.99	20	2.15	0.59	20	13.4%	0.20 [−0.31, 0.71]
Yuan 2020	1	1.54	40	3	3.07	40	11.2%	−2.00 [−3.06, −0.94]
**Subtotal (95% CI)**			**329**			**408**	**100.0%**	**−1.12 [−1.89, −0.34]**
Heterogeneity: $Tau^2 = 1.11$; $Chi^2 = 105.55$, df = 7 (P < 0.00001); $I^2 = 93\%$								
Test for overall effect: Z = 2.82 (P = 0.005)								
**1.2.4 24 hours**								
Abduallah 2020	1.33	2.34	30	1.33	2.34	30	8.6%	0.00 [−1.18, 1.18]
Brixel 2021	0.85	1.53	50	1.19	1.53	50	11.9%	−0.34 [−0.94, 0.26]
Haskins 2021	3.3	2.5	46	3.8	2.1	46	9.9%	−0.50 [−1.44, 0.44]
He 2018	2	0.5	30	3.3	1.1	30	12.8%	−1.30 [−1.73, −0.87]
He 2020	2.39	0.83	44	3.38	0.96	44	13.0%	−0.99 [−1.36, −0.62]
Kukreja 2019	4.56	2.47	79	4.22	2.63	159	11.5%	0.34 [−0.34, 1.02]
Kukreja1 2019	2.31	2.22	36	4.06	2.48	35	9.0%	−1.75 [−2.85, −0.65]
Tulgar 2018	2.1	0.45	20	1.95	0.39	29	13.5%	0.15 [−0.09, 0.39]
Yuan 2020	0.67	0.77	40	3	3.07	40	9.7%	−2.33 [−3.31, −1.35]
**Subtotal (95% CI)**			**375**			**463**	**100.0%**	**−0.71 [−1.27, −0.15]**
Heterogeneity: $Tau^2 = 0.59$; $Chi^2 = 71.51$, df = 8 (P < 0.00001); $I^2 = 89\%$								
Test for overall effect: Z = 2.49 (P = 0.01)								
**1.2.5 48 hours**								
He 2018	1.6	0.5	30	3	0.7	30	39.5%	−1.40 [−1.71, −1.09]
He 2020	1.56	0.5	44	2.38	0.73	44	0.0%	−0.82 [−1.08, −0.56]
Kukreja 2019	4.11	2.4	79	3.95	2.63	159	36.0%	0.16 [−0.51, 0.83]
Kukreja1 2019	3.33	2.58	36	4.11	3.78	35	24.6%	−0.78 [−2.29, 0.73]
**Subtotal (95% CI)**			**145**			**224**	**100.0%**	**−0.69 [−1.88, 0.50]**
Heterogeneity: $Tau^2 = 0.91$; $Chi^2 = 17.45$, df = 2 (P = 0.0002); $I^2 = 89\%$								
Test for overall effect: Z = 1.13 (P = 0.26)								

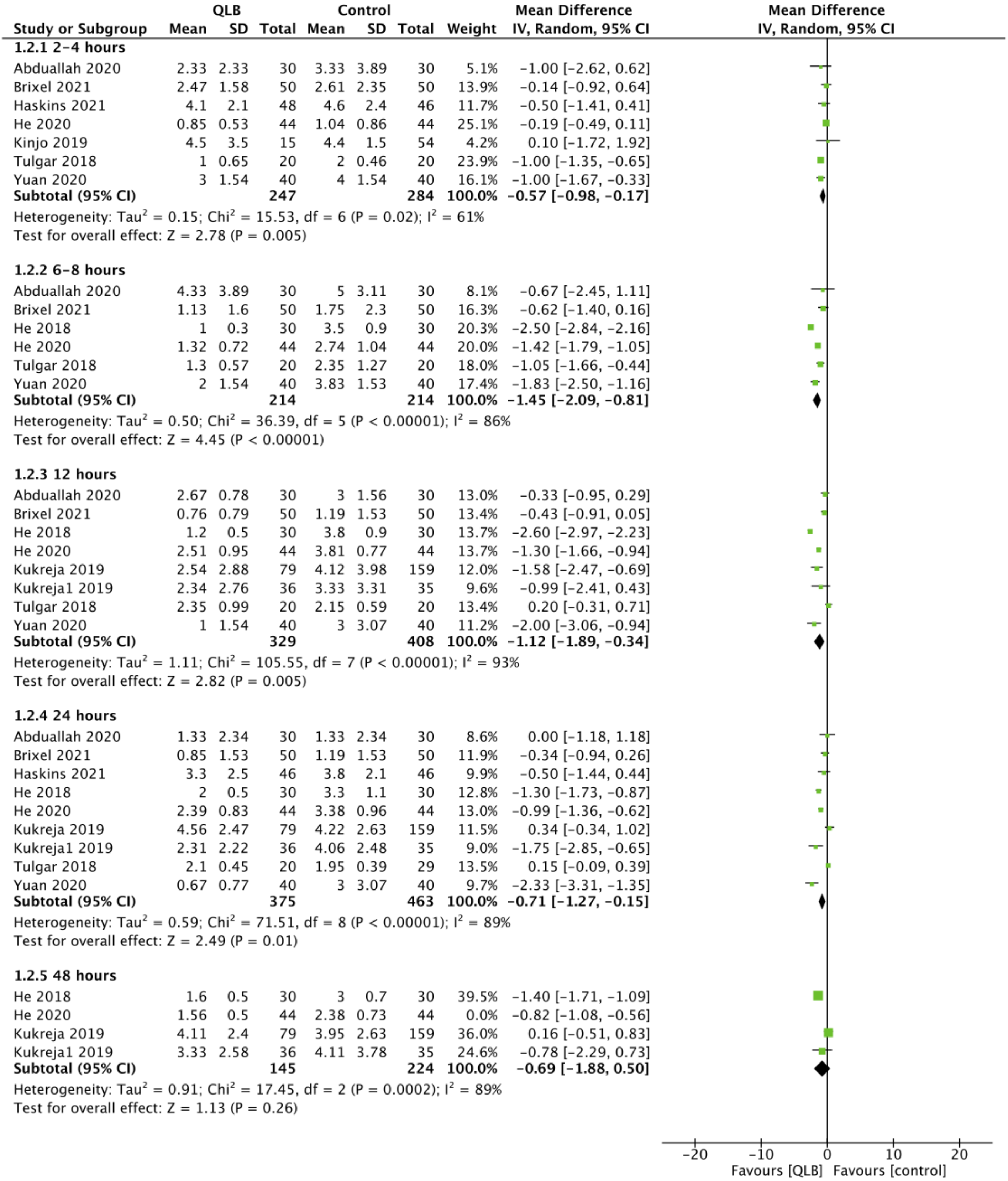

Supplementary figure 7

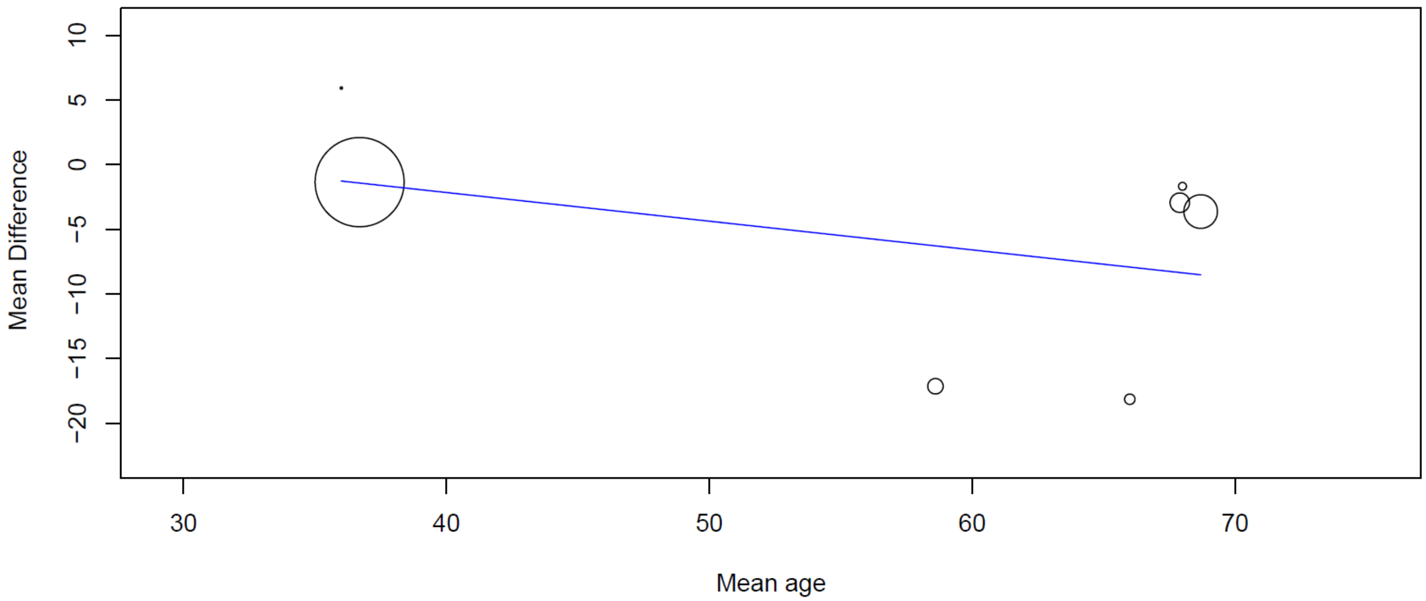

Supplementary figure 8

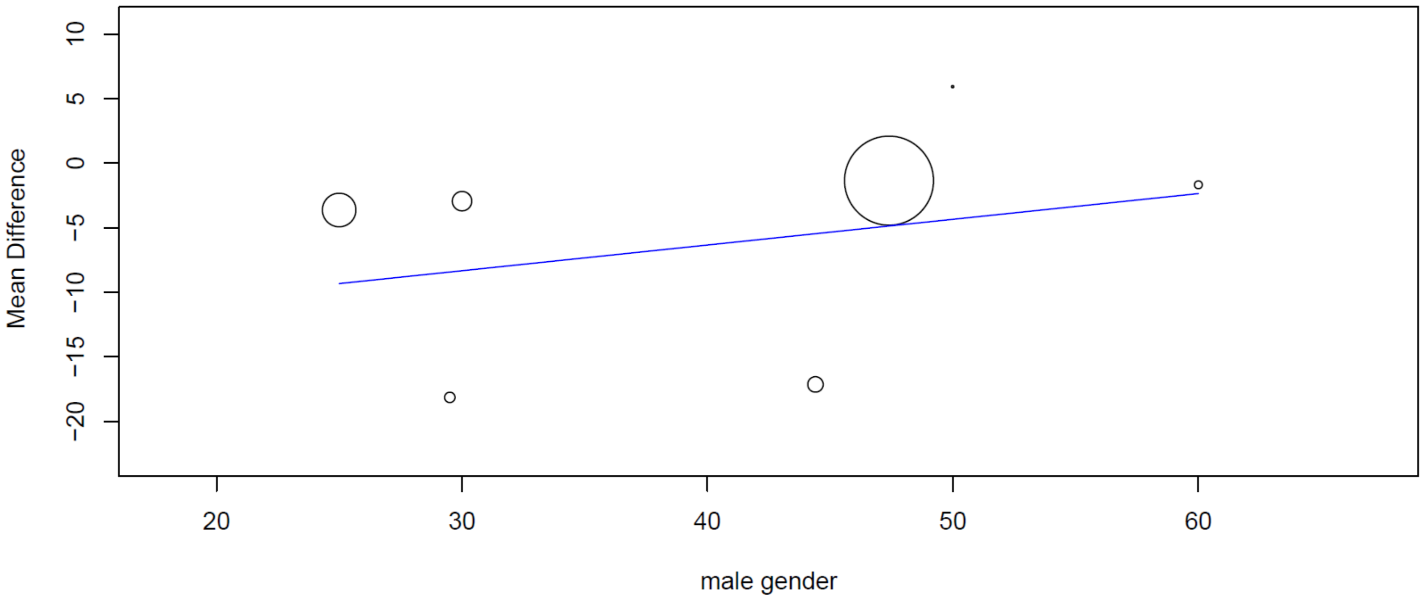

Supplementary figure 9

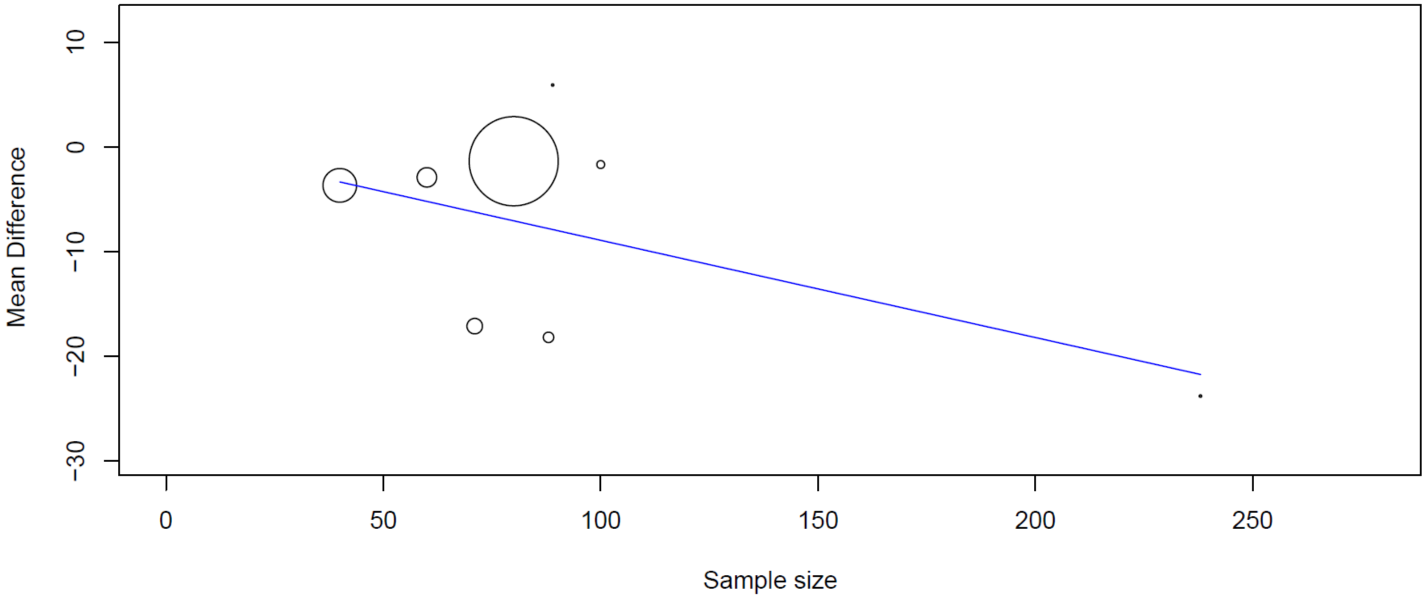

Supplementary figure 10

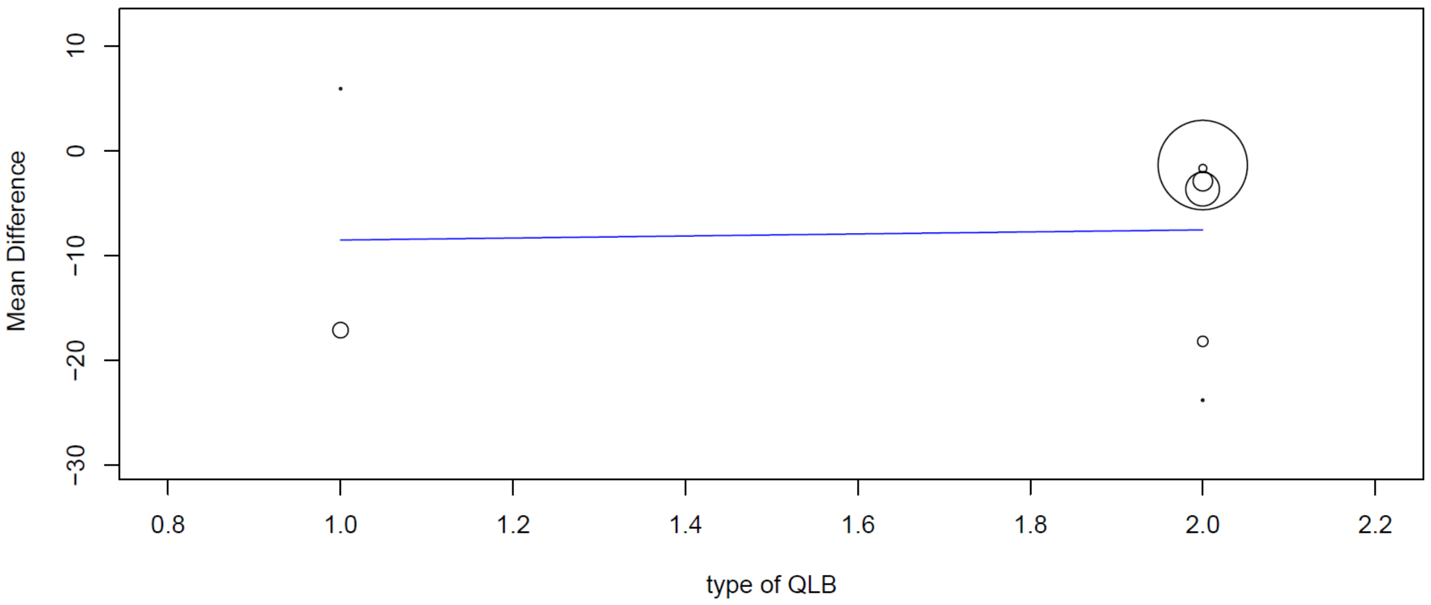

Supplementary figure 11

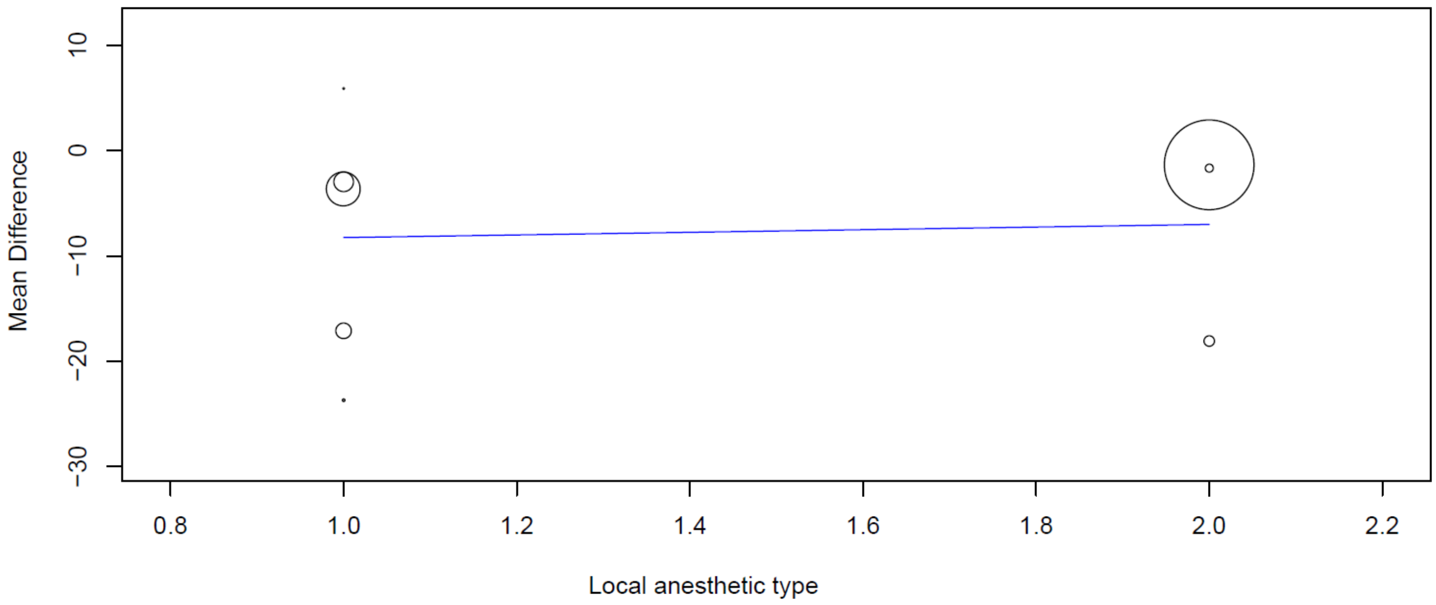

Supplementary figure 12

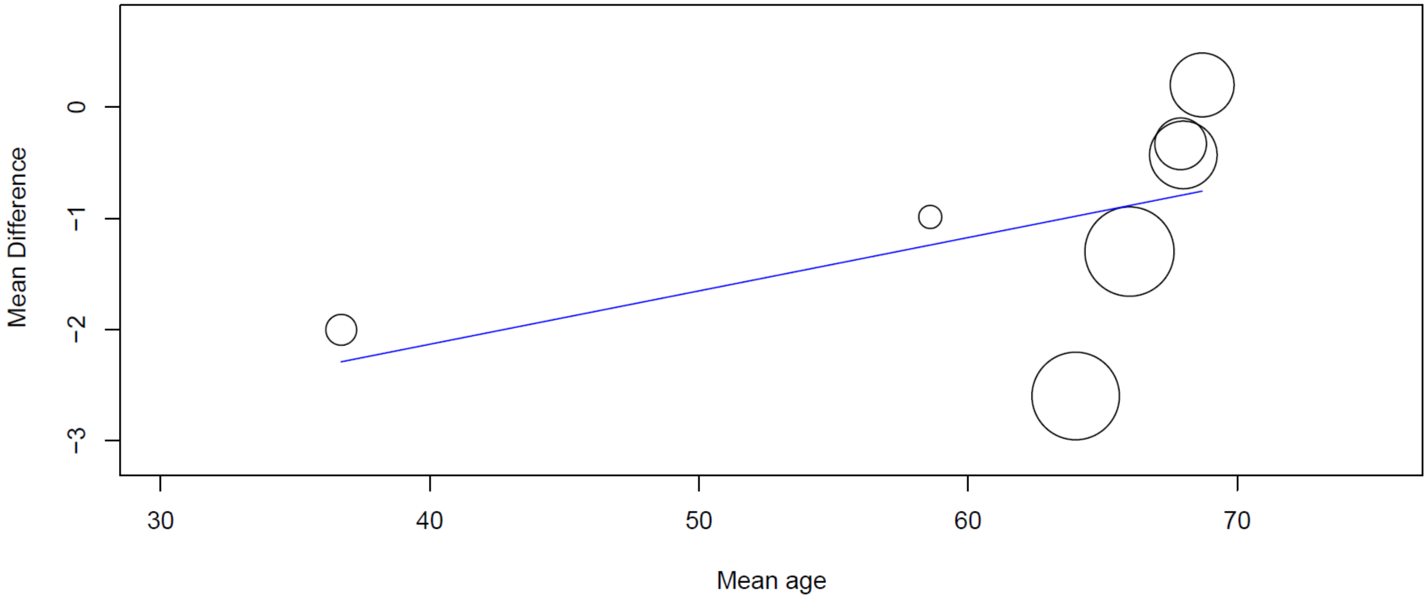

Supplementary figure 13

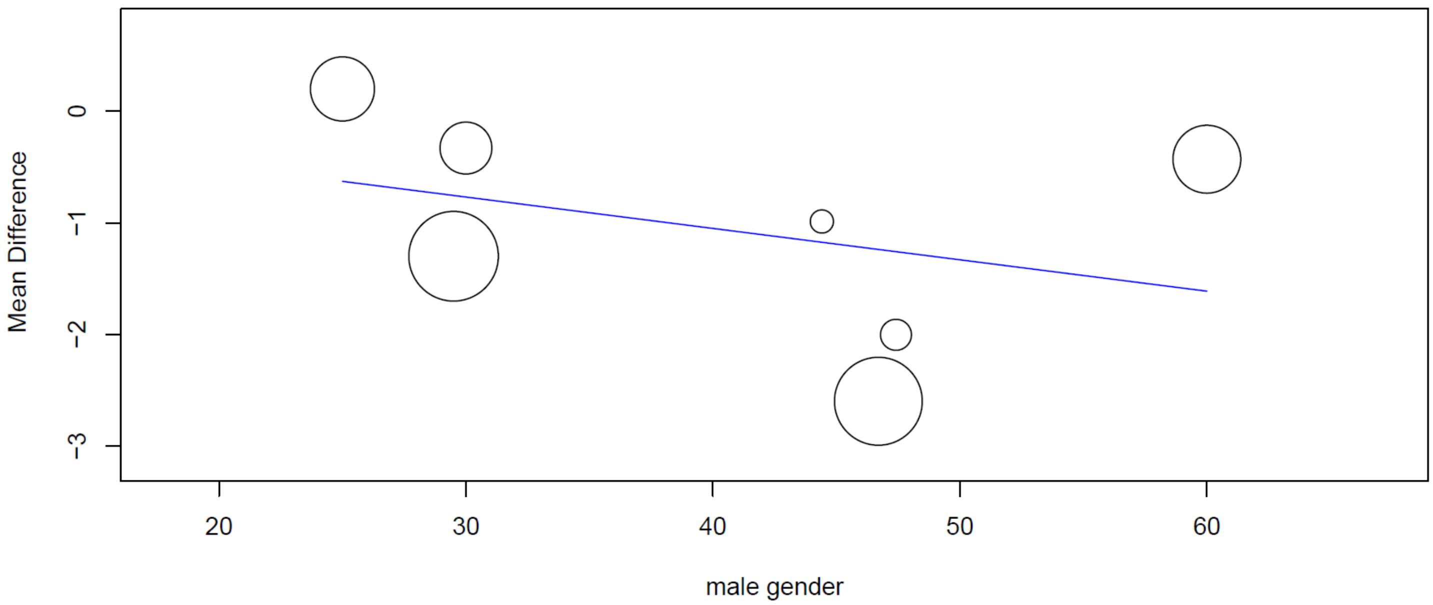

Supplementary figure 14

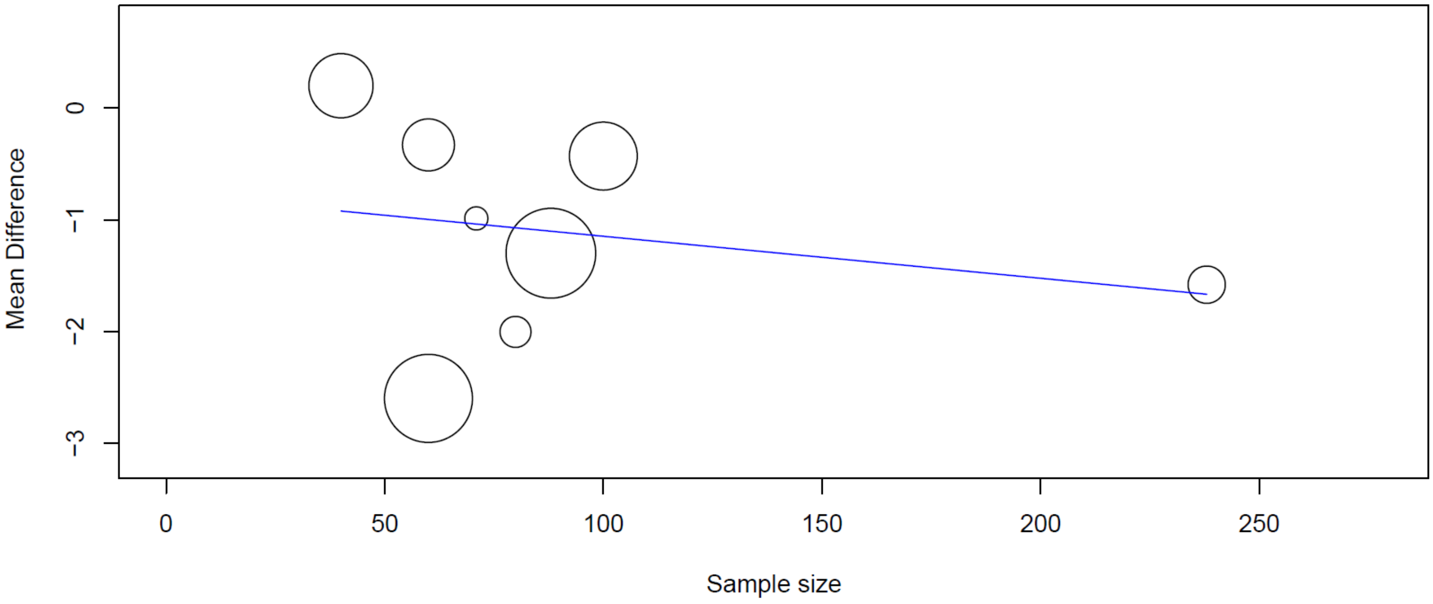

Supplementary figure 15

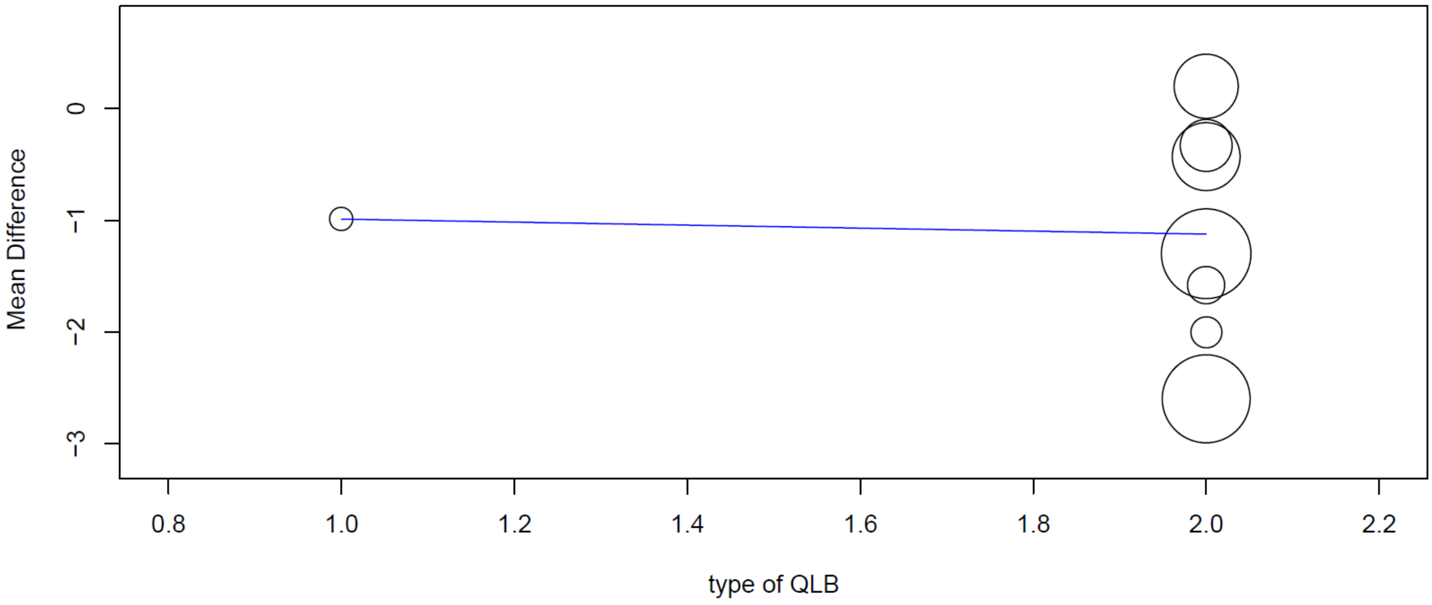

Supplementary figure 16

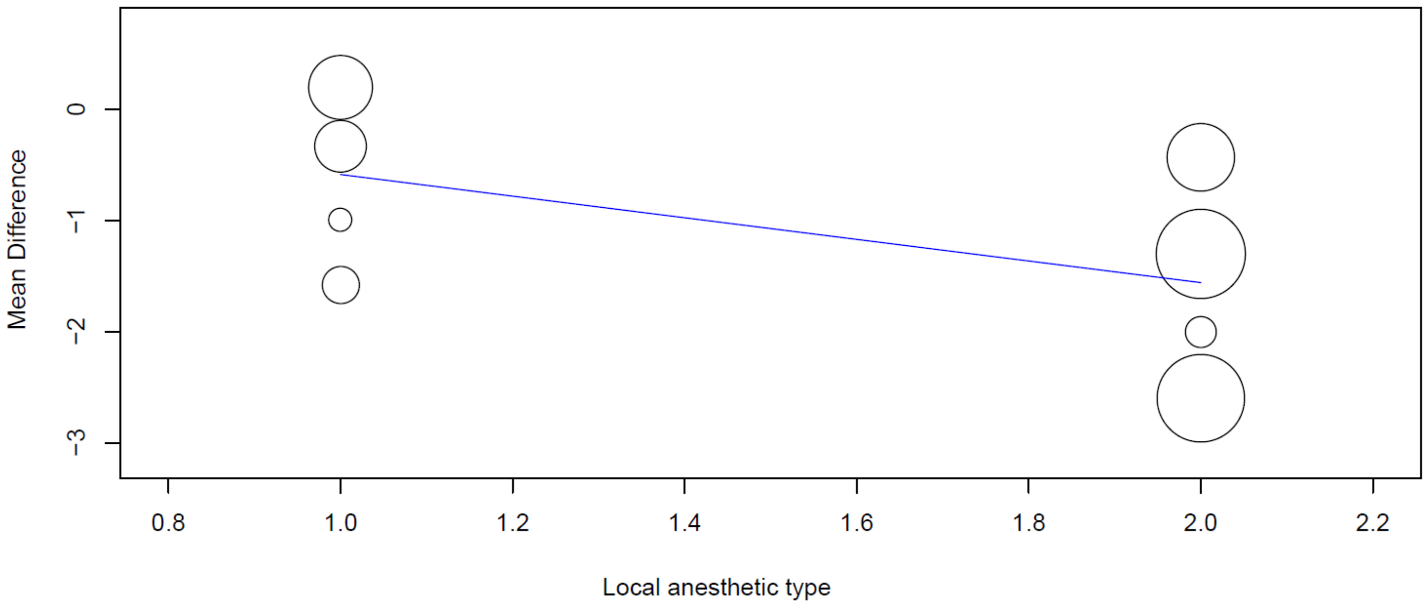

Supplementary figure 17

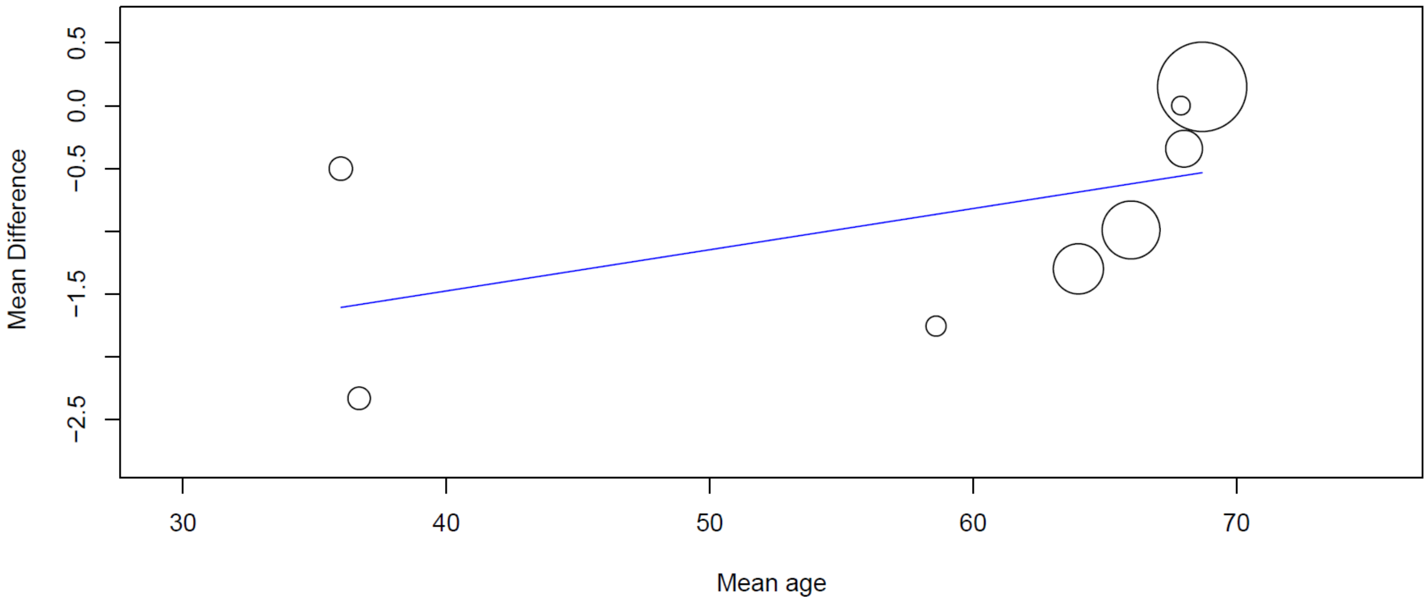

Supplementary figure 18

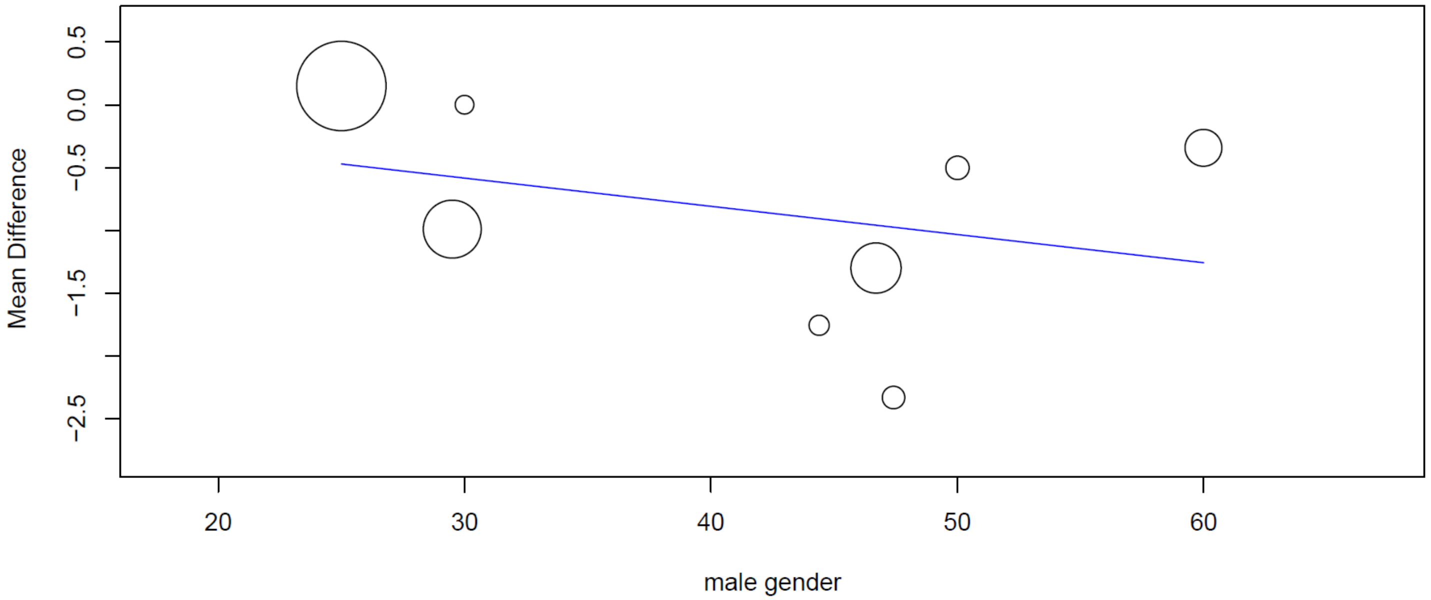

Supplementary figure 19

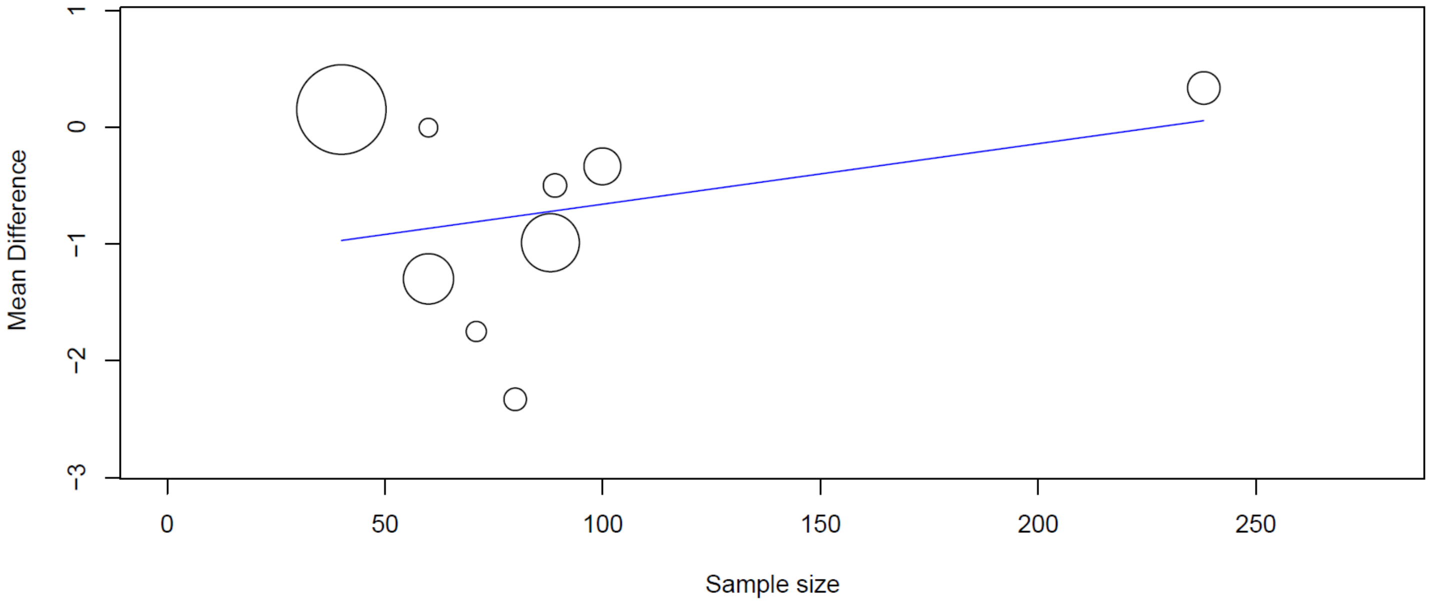

Supplementary figure 20

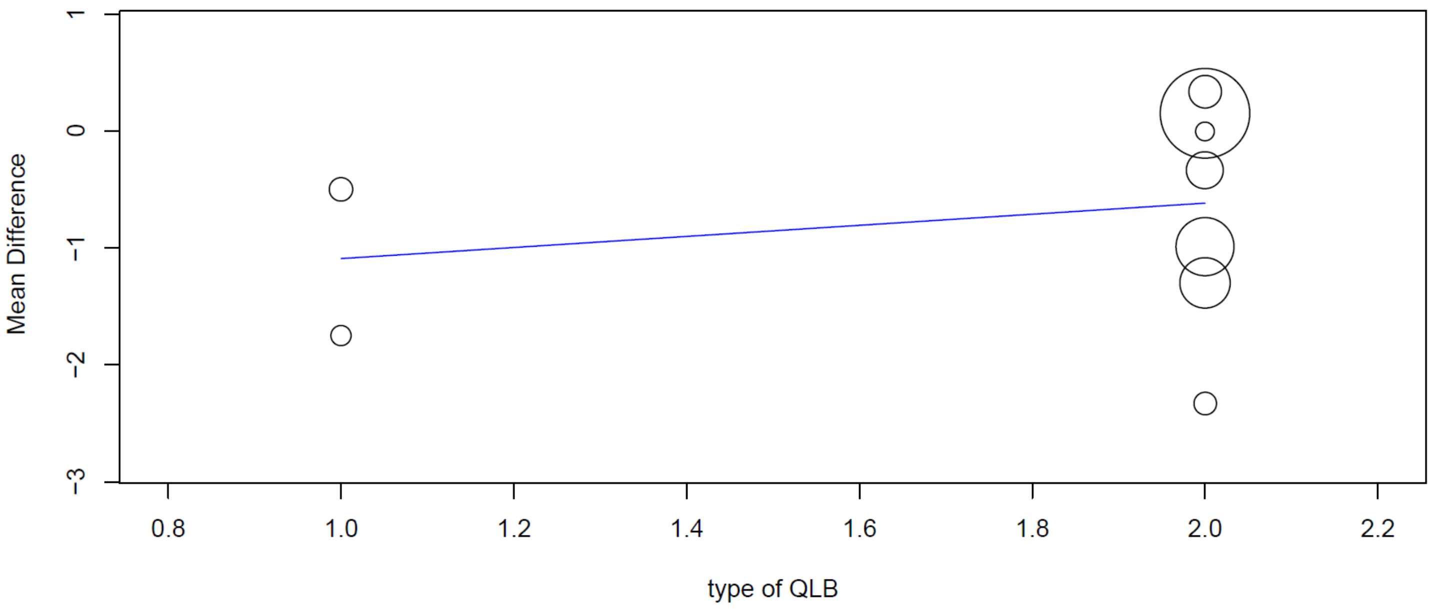

Supplementary figure 21

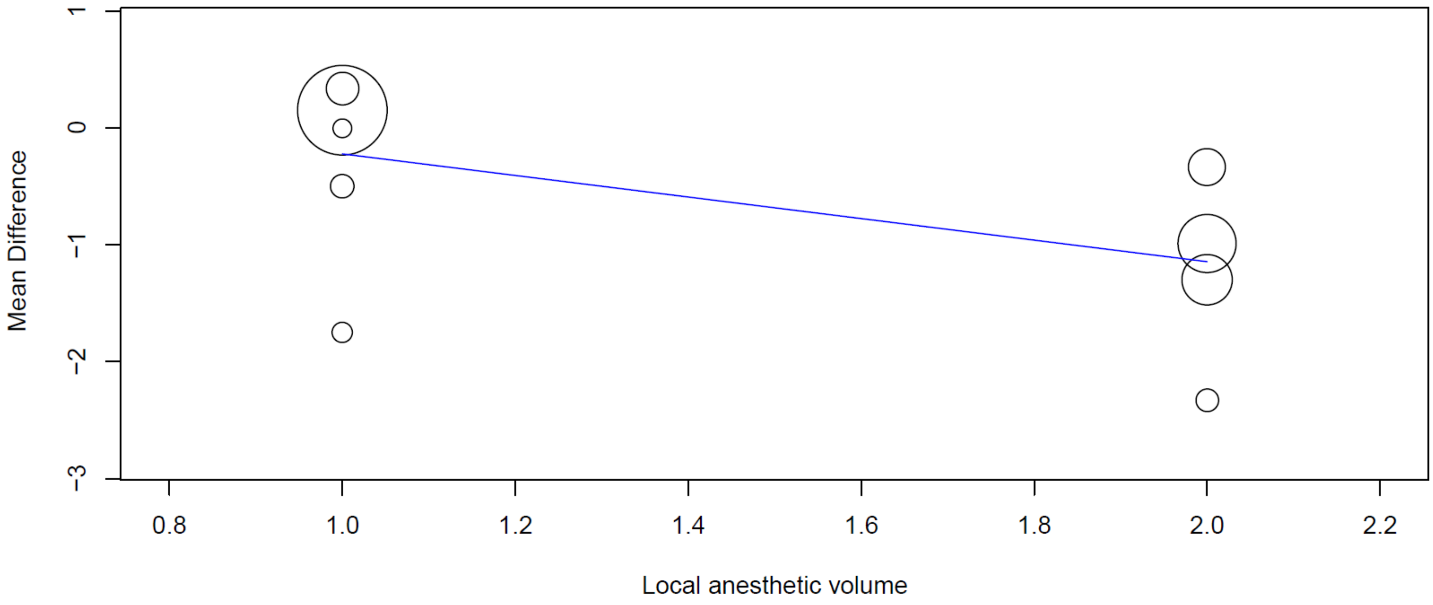

Supplement: Supplementary Figure 1 — Funnel plot for the meta-analysis of 24-h total analgesic consumption between QLB and control groups. [file Data_Sheet_1.PDF]
