# Supplementary material for: Efficacy of Quadratus Lumborum Block for Pain Control in Patients Undergoing Hip Surgeries: A Systematic Review and Meta-Analysis
Source: Front Med (Lausanne). 2022 Feb 3;8:771859. doi: 10.3389/fmed.2021.771859 (PMC8850973; doi:10.3389/fmed.2021.771859)
Supplement: Supplementary Table 2 — Search strategy. [file Table_2.DOCX]

Supplementary Table 2: Search strategy

| **Search number** | **Query** | **Search Details** |
| --- | --- | --- |
| **1** | (Quadratus lumborum block) AND (Hip surgery) | "Quadratus"[All Fields] AND "lumborum"[All Fields] AND ("block"[All Fields] OR "blocked"[All Fields] OR "blocking"[All Fields] OR "blockings"[All Fields] OR "blocks"[All Fields]) AND (("hip"[MeSH Terms] OR "hip"[All Fields]) AND ("surgery"[MeSH Subheading] OR "surgery"[All Fields] OR "surgical procedures, operative"[MeSH Terms] OR ("surgical"[All Fields] AND "procedures"[All Fields] AND "operative"[All Fields]) OR "operative surgical procedures"[All Fields] OR "general surgery"[MeSH Terms] OR ("general"[All Fields] AND "surgery"[All Fields]) OR "general surgery"[All Fields] OR "surgery s"[All Fields] OR "surgerys"[All Fields] OR "surgeries"[All Fields])) |
| **2** | (Quadratus lumborum block) AND (Hip fracture) | "Quadratus"[All Fields] AND "lumborum"[All Fields] AND ("block"[All Fields] OR "blocked"[All Fields] OR "blocking"[All Fields] OR "blockings"[All Fields] OR "blocks"[All Fields]) AND ("hip fractures"[MeSH Terms] OR ("hip"[All Fields] AND "fractures"[All Fields]) OR "hip fractures"[All Fields] OR ("hip"[All Fields] AND "fracture"[All Fields]) OR "hip fracture"[All Fields]) |
| **3** | (Quadratus lumborum block) AND (Hip arthroplasty) | "Quadratus"[All Fields] AND "lumborum"[All Fields] AND ("block"[All Fields] OR "blocked"[All Fields] OR "blocking"[All Fields] OR "blockings"[All Fields] OR "blocks"[All Fields]) AND (("hip"[MeSH Terms] OR "hip"[All Fields]) AND ("arthroplasty"[MeSH Terms] OR "arthroplasty"[All Fields] OR "arthroplasties"[All Fields])) |
| **4** | (Quadratus lumborum block) AND (Hip arthroscopy) | "Quadratus"[All Fields] AND "lumborum"[All Fields] AND ("block"[All Fields] OR "blocked"[All Fields] OR "blocking"[All Fields] OR "blockings"[All Fields] OR "blocks"[All Fields]) AND (("hip"[MeSH Terms] OR "hip"[All Fields]) AND ("arthroscopy"[MeSH Terms] OR "arthroscopy"[All Fields] OR "arthroscopies"[All Fields])) |
| **5** | (Quadratus lumborum block) AND (regional anesthesia) | "Quadratus"[All Fields] AND "lumborum"[All Fields] AND ("block"[All Fields] OR "blocked"[All Fields] OR "blocking"[All Fields] OR "blockings"[All Fields] OR "blocks"[All Fields]) AND ("regional anaesthesia"[All Fields] OR "anesthesia, conduction"[MeSH Terms] OR ("anesthesia"[All Fields] AND "conduction"[All Fields]) OR "conduction anesthesia"[All Fields] OR ("regional"[All Fields] AND "anesthesia"[All Fields]) OR "regional anesthesia"[All Fields]) |
