# Supplementary material for: Efficacy of Quadratus Lumborum Block for Pain Control in Patients Undergoing Hip Surgeries: A Systematic Review and Meta-Analysis
Source: Front Med (Lausanne). 2022 Feb 3;8:771859. doi: 10.3389/fmed.2021.771859 (PMC8850973; doi:10.3389/fmed.2021.771859)
Supplement: Supplementary Table 3 — GRADE assessment of evidence. [file Table_3.DOCX]

| **Supplementary Table 3: GRADE assessment of evidence** | | | | | | | | | | | |
| --- | --- | --- | --- | --- | --- | --- | --- | --- | --- | --- | --- |
| **Certainty assessment** | | | | | | | **Summary of findings** | | | | |
| **Participants  (studies) Follow up** | **Risk of bias** | **Inconsistency** | **Indirectness** | **Imprecision** | **Publication bias** | **Overall certainty of evidence** | **Study event rates (%)** | | **Relative effect (95% CI)** | **Anticipated absolute effects** | |
|  |  |  |  |  |  |  | **With placebo** | **With QLB** |  | **Risk with placebo** | **Risk difference with QLB** |
| **24 hour total opioid consumption** | | | | | | | | | | | |
| 528 (7 RCTs) | not serious | serious ^a^ | not serious | not serious | none | ⨁⨁⨁◯ MODERATE | 264 | 264 | - | The mean 24 hour total opioid consumption was **0** | MD **6.59 lower** (10.66 lower to 2.52 lower) |
| **pain scores - 2-4 hours** | | | | | | | | | | | |
| 462 (6 RCTs) | not serious | not serious | not serious | not serious | none | ⨁⨁⨁⨁ HIGH | 230 | 232 | - | The mean pain scores - 2-4 hours was **0** | MD **0.6 lower** (1.02 lower to 0.18 lower) |
| **pain scores - 6-8 hours** | | | | | | | | | | | |
| 428 (6 RCTs) | serious ^b^ | not serious | not serious | not serious | none | ⨁⨁⨁◯ MODERATE | 214 | 214 | - | The mean pain scores - 6-8 hours was **0** | MD **1.45 lower** (2.09 lower to 0.81 lower) |
| **pain scores - 12 hours** | | | | | | | | | | | |
| 499 (7 RCTs) | serious ^b^ | not serious | not serious | not serious | none | ⨁⨁⨁◯ MODERATE | 249 | 250 | - | The mean pain scores - 12 hours was **0** | MD **1.05 lower** (1.91 lower to 0.2 lower) |
| **pain scores - 24 hours** | | | | | | | | | | | |
| 600 (8 RCTs) | serious ^b^ | not serious | not serious | not serious | none | ⨁⨁⨁◯ MODERATE | 304 | 296 | - | The mean pain scores - 24 hours was **0** | MD **0.85 lower** (1.45 lower to 0.25 lower) |
| **pain scores - 48 hours** | | | | | | | | | | | |
| 219 (3 RCTs) | serious ^b^ | not serious | not serious | not serious | none | ⨁⨁⨁◯ MODERATE | 109 | 110 | - | The mean pain scores - 48 hours was **0** | MD **1.07 lower** (1.57 lower to 0.57 lower) |
| **Complications - PONV** | | | | | | | | | | | |
| 481 (6 RCTs) | serious ^b^ | not serious | not serious | serious ^c^ | none | ⨁⨁◯◯ LOW | 64/239 (26.8%) | 28/242 (11.6%) | **RR 0.40** (0.18 to 0.88) | 268 per 1,000 | **161 fewer per 1,000** (from 220 fewer to 32 fewer) |
| **Complications - Pruritis** | | | | | | | | | | | |
| 288 (4 RCTs) | serious ^b^ | not serious | not serious | serious ^c^ | none | ⨁⨁◯◯ LOW | 17/144 (11.8%) | 7/144 (4.9%) | **RR 0.46** (0.17 to 1.24) | 118 per 1,000 | **64 fewer per 1,000** (from 98 fewer to 28 more) |
| **Complications - Urinary retention** | | | | | | | | | | | |
| 284 (3 RCTs) | not serious | not serious | not serious | serious ^c^ | none | ⨁⨁⨁◯ MODERATE | 16/142 (11.3%) | 7/142 (4.9%) | **RR 0.44** (0.19 to 1.02) | 113 per 1,000 | **63 fewer per 1,000** (from 91 fewer to 2 more) |

**CI:** Confidence interval; **MD:** Mean difference; **RR:** Risk ratio

#### Explanations

a. Significantly high heterogeneity in the meta-analysis

b. High risk of bias in the trial of He 2018

c. Limited number of studies in the analysis
