# Supplementary material for: Efficacy of Quadratus Lumborum Block for Pain Control in Patients Undergoing Hip Surgeries: A Systematic Review and Meta-Analysis
Source: Front Med (Lausanne). 2022 Feb 3;8:771859. doi: 10.3389/fmed.2021.771859 (PMC8850973; doi:10.3389/fmed.2021.771859)
Supplement: Supplementary Table 4 — Meta-regression analysis for the heterogeneity of post-operative 24 h total analgesic consumption. [file Table_4.DOCX]

Supplementary Table 4. Meta-regression analysis for the heterogeneity of postoperative 24 hour total analgesic consumption.

| Variances | Coefficient | SE | 95% CI | P value | Scatter plot |
| --- | --- | --- | --- | --- | --- |
| Mean age | -0.22 | 0.21 | -0.64 to 0.19 | 0.30 | Supplementary figure 7 |
| Male gender | 0.19 | 0.23 | -0.25 to 0.65 | 0.39 | Supplementary figure 8 |
| Sample size | -0.09 | 0.02 | -0.20 to 0.01 | 0.09 | Supplementary figure 9 |
| QLB type | 0.96 | 7.24 | -13.22 to 15.16 | 0.89 | Supplementary figure 10 |
| Bupivacaine vs Ropivacaine | 1.25 | 6.15 | -10.81 to 13.31 | 0.83 | Supplementary figure 11 |

Abbreviations: QLB, quadratus lumborum block; SE, standard error; CI, confidence interval
