# Supplementary material for: Efficacy of Quadratus Lumborum Block for Pain Control in Patients Undergoing Hip Surgeries: A Systematic Review and Meta-Analysis
Source: Front Med (Lausanne). 2022 Feb 3;8:771859. doi: 10.3389/fmed.2021.771859 (PMC8850973; doi:10.3389/fmed.2021.771859)
Supplement: Supplementary Table 5 — Meta-regression analysis for the heterogeneity of 12 h pain scores. [file Table_5.DOCX]

Supplementary Table 5. Meta-regression analysis for the heterogeneity of 12 hours pain scores.

| Variances | Coefficient | SE | 95% CI | P value | Scatter plot |
| --- | --- | --- | --- | --- | --- |
| Mean age | 0.03 | 0.21 | -0.01 to 0.11 | 0.14 | Supplementary figure 12 |
| Male gender | -0.02 | 0.3 | -0.08 to 0.02 | 0.32 | Supplementary figure 13 |
| Sample size | -0.00 | 0.01 | -0.02 to 0.01 | 0.51 | Supplementary figure 14 |
| QLB type | -0.14 | 1.17 | -2.43 to 2.16 | 0.90 | Supplementary figure 15 |
| Bupivacaine vs Ropivacaine | -0.97 | 0.56 | -2.08 to 0.14 | 0.09 | Supplementary figure 16 |

Abbreviations: QLB, quadratus lumborum block; SE, standard error; CI, confidence interval
