# Supplementary material for: Efficacy of Quadratus Lumborum Block for Pain Control in Patients Undergoing Hip Surgeries: A Systematic Review and Meta-Analysis
Source: Front Med (Lausanne). 2022 Feb 3;8:771859. doi: 10.3389/fmed.2021.771859 (PMC8850973; doi:10.3389/fmed.2021.771859)
Supplement: Supplementary Table 6 — Meta-regression analysis for the heterogeneity of 24 h pain scores. [file Table_6.DOCX]

Supplementary Table 6. Meta-regression analysis for the heterogeneity of 24 hours pain scores.

| Variances | Coefficient | SE | 95% CI | P value | Scatter plot |
| --- | --- | --- | --- | --- | --- |
| Mean age | 0.03 | 0.02 | -0.01 to 0.07 | 0.09 | Supplementary figure 17 |
| Male gender | -0.02 | 0.02 | -0.06 to 0.02 | 0.29 | Supplementary figure 18 |
| Sample size | 0.01 | 0.01 | -0.00 to 0.01 | 0.25 | Supplementary figure 19 |
| QLB type | 0.47 | 0.68 | -0.86 to 1.82 | 0.49 | Supplementary figure 20 |
| Bupivacaine vs Ropivacaine | -0.92 | 0.41 | -1.73 to -0.11 | 0.02 | Supplementary figure 21 |

Abbreviations: QLB, quadratus lumborum block; SE, standard error; CI, confidence interval
